# KIT supports small intestinal tuft cell hyperplasia

**DOI:** 10.1126/sciadv.ady0883

**Published:** 2026-02-25

**Authors:** Heber I. Lara, Madeleine R. Bell, Shealyn O’Connor, Hung-An Ting, Jakob von Moltke

**Affiliations:** Department of Immunology, University of Washington School of Medicine, Seattle, WA, USA.

## Abstract

The small intestine balances the competing tasks of nutrient absorption, immune tolerance, and defense through dynamic differentiation of short-lived epithelial cells. During helminth infection, interleukin-13 (IL-13) or IL-4 drive a 10-fold expansion of tuft cells to promote helminth clearance. While IL-4/13 signaling in epithelial cells is required for tuft cell hyperplasia, few signals that support this process have been identified. Here, we show that tuft cells across all tissues express the receptor tyrosine kinase KIT and that IL-4/13 is necessary and sufficient to up-regulate KIT on small intestinal (SI) tuft cells. Although epithelial KIT is dispensable for homeostatic turnover, KIT deletion from tuft cells during helminth infection reduces tuft cell hyperplasia and delays helminth clearance. Mechanistically, KIT signaling supports the generation of new tuft cells in SI crypts. These findings thus identify a unique tuft cell-specific function for KIT in type 2 immunity.

## Teaser

Small intestinal type 2 inflammation up-regulates KIT on tuft cells to promote tuft cell hyperplasia and helminth clearance.

## INTRODUCTION

The small intestinal epithelium (SIE) is a single-cell layer that separates the intestinal lumen from the lamina propria and mediates nutrient absorption, microbial tolerance, and exclusion of pathogens. Balancing these diverse functions requires careful coordination of cellular differentiation that tunes the cellular composition and, thus, the function of the SIE. During type 2 immune responses triggered by helminth infection, interleukin-4 (IL-4) and/or IL-13 drive a 10-fold expansion of the epithelial tuft cell lineage that is required for immune protection (*1*, *2*). Despite the magnitude of this tissue remodeling, the mechanisms that initiate and sustain tuft cell hyperplasia remain poorly defined.

The SIE is folded into two anatomically distinct repeated cellular compartments: small, pocket-like crypts and large villi that extend into the lumen. Stem and progenitor cells reside within the crypts where they proliferate and replenish the surrounding villi (*3*, *4*). The SIE is populated by five major cell types, each of which can be assigned to the absorptive or secretory lineage. Absorptive enterocytes facilitate nutrient and fluid uptake and comprise about 80% of the SIE at homeostasis (*5*). Secretory enteroendocrine, Paneth, goblet, and tuft cells are defined by their specialized secreted products such as hormones, mucus, and cytokines (*4*).

Cell division and differentiation occur in the crypts, after which most lineages migrate to the villus tips and are expelled into the lumen. Paneth cells are the exception as they remain at the crypt base (*6*). The balance between absorptive and secretory cells is largely determined by growth and patterning factors that direct the daily proliferation, differentiation, and maintenance of the five major cell lineages. Wnt and epidermal growth factor (EGF) drive cell division at the crypt base, while bone morphogenetic protein (BMP) antagonizes Wnt signaling to allow cellular differentiation and specialization in the upper crypt region (*7*, *8*). Other growth factors including glucagon-like peptide 2 and granulocyte colony stimulating factor have been linked to epithelial repair and can be induced by chemical or mechanical stress (*9*).

The small intestinal (SI) villus epithelium is replaced every 3 to 5 days, allowing for rapid adaptations through shifts in cellular composition (*4*). For example, during parasitic worm (helminth) infection, the frequency of goblet and tuft cells increases, the latter by as much as 10-fold (*1*, *2*). Goblet cells secrete mucus and RELMβ to reduce helminth attachment and fitness (*10*, *11*). Tuft cells, commonly identified by their expression of doublecortin-like kinase 1 (DCLK1), function as both sentinels that detect helminths in the lumen and, once tuft cell hyperplasia occurs, as effectors that help to expel helminths from the intestine (*1*, *2*, *12*, *13*).

Both skin-penetrating hookworms, such as *Nippostrongylus brasiliensis*, and orally transmitted helminths, such as *Heligmosomoides polygyrus bakeri*, settle in the SI lumen, where they mature and mate (*10*). Here, tuft cells sense the worms and initiate type 2 immunity by secreting IL-25 and leukotriene C_4_ (LTC_4_) to activate group 2 innate lymphoid cells (ILC2s) in the SI lamina propria (*1*, *2*, *14*). Activated ILC2s secrete IL-13, which signals in crypt epithelial cells to promote differentiation into more tuft and goblet cells, thus completing a feed-forward “tuft-ILC2 circuit.” Tuft cell (and type II taste receptor cell) differentiation selectively depends on the transcription factor POU2F3 as *Pou2f3^−/−^* mice lack all tuft cells, but nontuft epithelial lineages are unaffected (*1*, *15*). In these mice, SI remodeling and type 2 inflammation are reduced, and worm clearance is delayed.

How tuft cells sense helminths remains unknown, but during colonization with *Tritrichomonas* protists and in certain states of bacterial dysbiosis, the tuft-ILC2 circuit is activated when tuft cells sense luminal accumulation of succinate (*16*–*19*). Tuft cell hyperplasia can also be induced by injecting recombinant IL-25 to directly activate ILC2s or by administering succinate in drinking water to directly activate tuft cells (*2*, *17*–*19*). In all cases, tuft cell hyperplasia is dependent on IL-4 or IL-13 signaling directly on epithelial cells. These cytokines use a shared heterodimeric receptor consisting of IL-4RA and IL-13RA1 and are sufficient to induce tuft cell hyperplasia in SI-derived organoids (enteroids) (*2*, *16*, *18*, *20*). Consistently, tuft cell hyperplasia does not occur when mice lack *Il4ra* selectively in epithelial cells (*2*). BMP and butyrate can restrain tuft cell hyperplasia, while epithelial RANK signaling promotes tuft cell differentiation (*21*–*23*). Other signals that act on the epithelium to support IL-4/13–induced tuft cell hyperplasia have not been identified.

KIT (c-Kit/CD117) is a transmembrane receptor tyrosine kinase (RTK) that supports cell growth, function, and survival. Following dimerization of KIT by its ligand, stem cell factor (SCF), transphosphorylation at tyrosine residues activates multiple signaling pathways including the phosphatidylinositol 3-kinase (PI3K) signaling cascade (*24*–*26*). KIT is expressed by both stem and differentiated cells across multiple tissue types and organs. In the small intestine, it is constitutively expressed by neuronal pacemaker cells (interstitial cells of Cajal) and Paneth cells (*27*, *28*). KIT has been shown to act as a regenerative factor in dextran sulfate sodium–induced colitis, during which it is thought to induce stem cell–like reprograming of Paneth cells (*29*). Some colonic crypt epithelial cells lacking the Wnt receptor and stem cell marker LGR5 also express KIT and can repopulate the epithelium, especially in regenerative states (*30*).

Mast cells, key effectors of type 2 immunity, rely on KIT, as demonstrated by the lack of mast cells in mice with hypomorphic *Kit* alleles (e.g., *Kit^W-sh/W-sh^*) (*31*). Type 2 immunity defects in *Kit^W-sh/W-sh^* mice have generally been interpreted to result from this lack of mast cells, but the pleiotropic functions of KIT make these conclusions difficult in the context of germline mutations that affect all cells (*32*–*36*). *Kit* expression has also been noted in mouse and human tuft cells, but its function in this context remains unclear (*37*, *38*). Here, we generated KIT floxed mice (“KIT10”) to characterize the SIE-specific role of KIT. We show that while epithelial KIT is dispensable for SIE homeostasis, during a type 2 immune response, it is up-regulated by IL-4/13 and required in committed tuft cells to achieve full tuft cell hyperplasia.

## RESULTS

### Tuft cells express KIT systemically at homeostasis

**Fig. 1.**
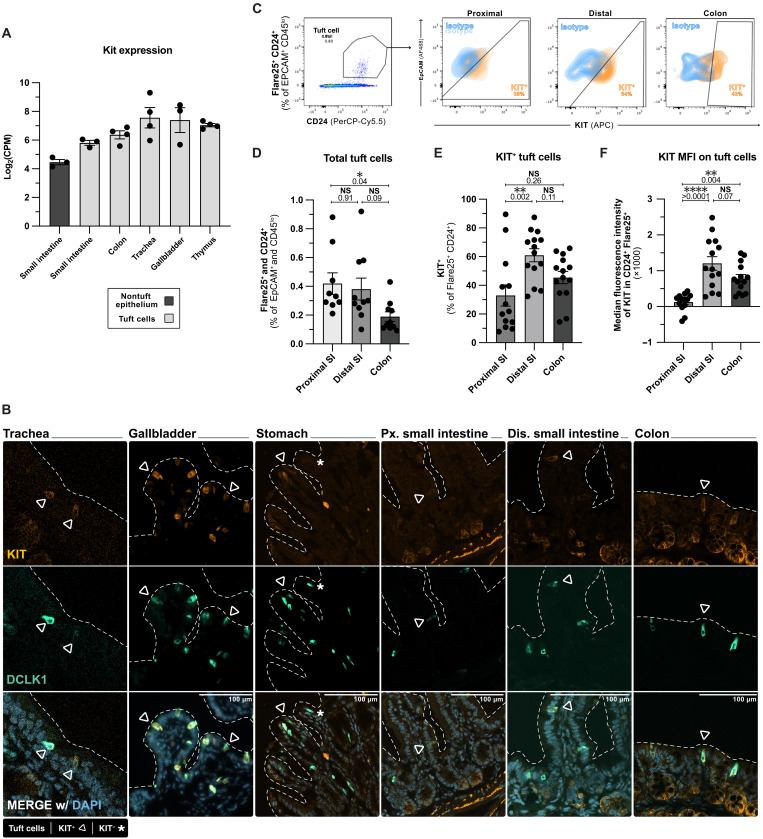
Tuft cells express KIT systemically at homeostasis. (**A**) Normalized RNA transcript reads in tuft cells and nontuft epithelium sorted from indicated tissue of unmanipulated Flare25 (*Il25^RFP/RFP^*) mice. Adapted from (*18*). (**B**) Immunofluorescent images of indicated tissues from unmanipulated wildtype C57BL/6 mice stained as indicated. (**C** to **F**) Flow cytometry analysis of intestinal epithelium taken from the proximal 5 cm of small intestine, distal 5 cm of small intestine, and proximal 5 cm of colon of unmanipulated Flare25 (*Il25^RFP/RFP^*) mice. (C) Representative tuft cell gating and KIT expression. (D) Total tuft cell frequency. (E) Frequency of KIT^+^ tuft cells. (F) Median fluorescent intensity (MFI) of KIT among tuft cells. MFI of the tissue isotype control was subtracted from the sample. In graphs, each data point represents a biological replicate. Data are pooled from at least two experiments [(A) and (D) to (F)] or representative of at least three experiments [(B) and (C)]. Statistics: ordinary one-way analysis of variance (ANOVA) [(D) to (F)]. DAPI, 4′,6-diamidino-2-phenylindole; PerCP, peridinin-chlorophyll-protein; NS, not signficant; AF488, Alexa Fluor 488; APC, allophycocyanin; EpCAM, epithelial cell adhesion molecule.

Having previously identified IL-4/13 signaling and alternative splicing of *Pou2af2* as SIE-intrinsic regulators of tuft cell differentiation and hyperplasia (*2*, *39*), we searched for additional regulators of these processes. We and others noted *Kit* expression in datasets from human and murine tuft cells as well as tuft cell–like carcinomas (*38*, *40*–*42*). The reanalysis of our previously published bulk RNA sequencing data revealed *Kit* expression in homeostatic tuft cells across all tissues surveyed, with the highest transcript reads in the gallbladder and trachea and the lowest in the small intestine (SI) and its nontuft epithelium (Fig. 1A) (*18*). Immunofluorescence labeling confirmed KIT protein expression on at least some tuft cells (identified as DCLK1^+^) in all tissues tested (Fig. 1B). We also confirmed previous reports that some DCLK1^−^ cells in the colonic crypt epithelium express KIT (*28*, *30*).

Given the critical role of SI tuft cells in helminth infection, their 10-fold expansion during SI type 2 inflammation, and a body of literature linking KIT to antihelminth immunity (*32*, *34*), we focused further analysis on SIE KIT. Consistent with prior reports, we found that Paneth cells constitutively express KIT, whereas LGR5^+^ stem cells do not (fig. S1A) (*29*). Flow cytometry analysis revealed that proximal (defined here as the first 5 to 12 cm from the stomach) and distal (last 5 to 12 cm before the cecum) regions of the SIE contained similar frequencies of tuft cells, but KIT expression was significantly higher in distal tuft cells by proportion and per-cell expression (Fig. 1, C to F). Together, a subset of tuft cells homeostatically expresses KIT protein in all tissues examined.

### Epithelial KIT is dispensable in the homeostatic small intestine

**Fig. 2.**
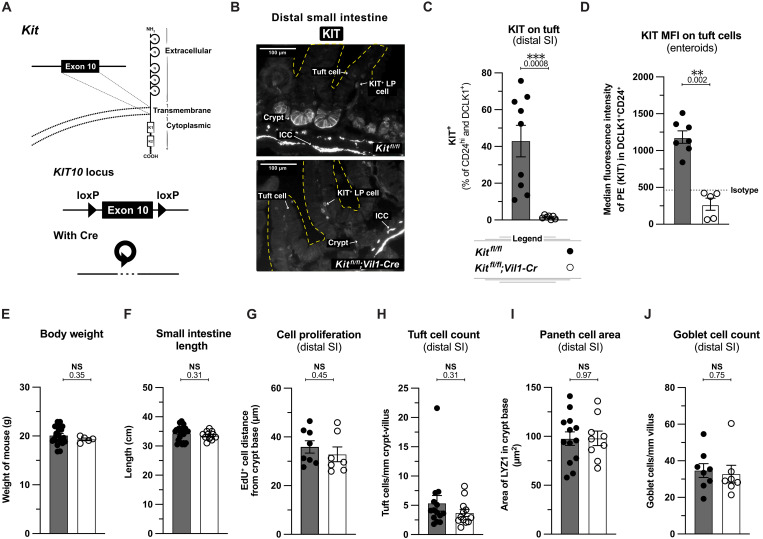
Epithelial KIT is dispensable in the homeostatic small intestine. (**A**) Schematic of Cre-Lox targeting of *Kit*. (**B**) Immunofluorescent (IF) imaging of KIT in distal (last 10 cm) small intestine from unmanipulated mice of indicated genotypes. ICC, interstitial cell of Cajal; LP, lamina propria. (**C**) Flow cytometric quantification of KIT on tuft cells (DCLK1^+^ CD24^+^) relative to an isotype control for KIT. Data from distal small intestine (10 cm section before the cecum) of unmanipulated mice of indicated genotypes. (**D**) Flow cytometric quantification of KIT median fluorescence intensity (MFI) on tuft cells from proximal (first 10 cm) small intestine–derived enteroids maintained in culture for 7 days. (**E** to **J**) Analysis of indicated parameters in unmanipulated *Kit^fl/fl^* and *Kit^fl/fl^;Vil1-Cre* littermates. In (G), mice were injected intraperitoneally with EdU 17 to 20 hours before analysis. In (H), tuft cells were identified by IF staining for DCLK1. In (I), Paneth cells were identified by IF staining for LYZ1. In (J), villus goblet cells were identified by IF staining with WGA. In graphs, each data point represents a biological replicate. Data are representative of three experiments (B) or pooled from at least two experiments [(C) to (J)]. Statistics: unpaired *t* test [(C) to (J)]. PE, phycoerythrin.

KIT inhibitors and mice carrying loss of function *Kit* mutations have demonstrated that KIT contributes to the division, migration, and/or survival of many cells (*36*). For example, stem-like function of Paneth cells was reduced in mice treated with a KIT inhibitor (*29*). One limitation of these studies, however, is the indiscriminate and/or incomplete targeting of KIT. To genetically test the function of KIT specifically in the SIE, we used CRISPR-Cas9 editing of mouse embryos to insert *loxP* sites flanking exon 10, which encodes the transmembrane domain of KIT (Fig. 2A and fig. S2A). We call this the “KIT10” mouse line. We first crossed KIT10 mice with *Vil1-Cre* mice to delete KIT from SIE stem cells and all differentiated progeny. We validated the deletion by immunofluorescence staining of homeostatic SI tissue and flow cytometry analysis of tuft cells from the small intestine and small intestine-derived enteroids (Fig. 2, B to D, and fig. S2B). Mice lacking SIE KIT had no difference in body weight, SI length, and overnight 5-ethynyl-2′-deoxyuridine (EdU) uptake (Fig. 2, E to G). Furthermore, *Vil1-*Cre^+^ KIT10 mice had no homeostatic defects in secretory cell differentiation as tuft, Paneth, and goblet cell frequencies were identical to wild-type littermates (Fig. 2, H to J). Thus, despite constitutive expression, SIE KIT is dispensable for SI function, cellular turnover, and differentiation at homeostasis.

### IL-4/13 is necessary and sufficient to induce KIT on small intestinal tuft cells

Given the central role of IL-4/13 in tuft cell expansion (*2*) and the high transcript expression of IL-4/13 receptor (*Il13ra1* + *Il4ra*) in tuft cells (*40*), we tested whether IL-4/13 modulates KIT on tuft cells. We first bulk-sequenced SI tuft cells from mice with tuft cell–specific deletion of IL-4/13 receptors (*Il4ra*^fl/fl^;*Pou2f3*^Cre-ERT2/+^) and littermate controls. All mice were given tamoxifen 6 days before harvest to activate CRE function and stimulated with recombinant IL-4 complexed with anti–IL-4 antibody for the final 8 hours to enhance differences between the two groups (fig. S3A). *Kit* was the most significantly reduced transcript in the Cre^+^ (*Il4ra-*negative) tuft cells, suggesting up-regulation of *Kit* by IL-4RA/IL-13RA1 signaling in wild-type tuft cells (fig. S3, B and C, and table S1).

**Fig. 3.**
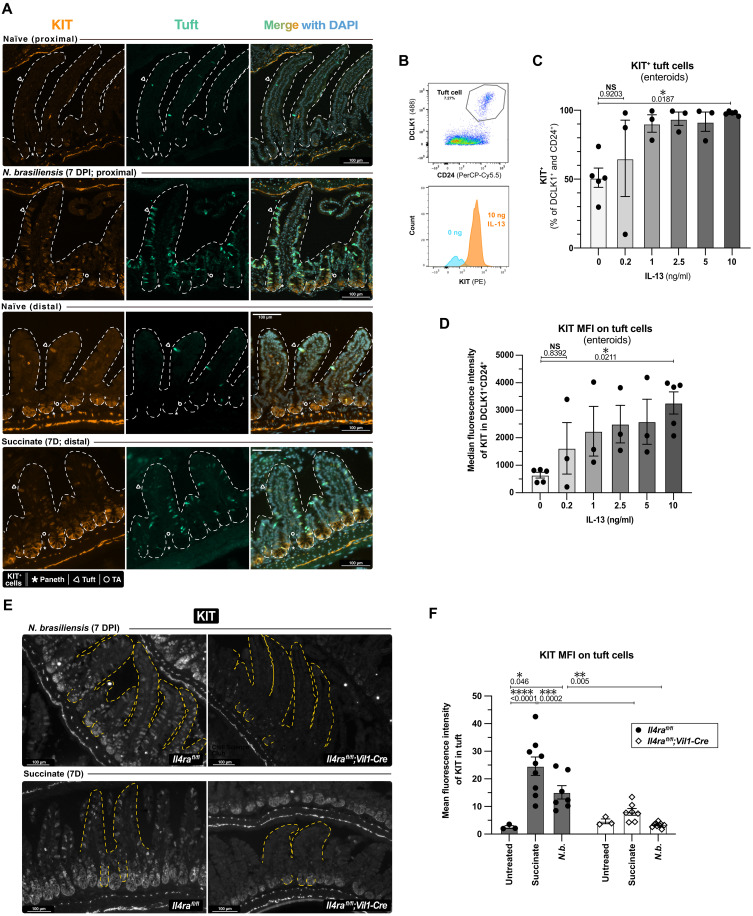
IL-4/13 is necessary and sufficient to induce KIT on small intestinal tuft cells. (**A**) Immunofluorescent staining for indicated proteins in small intestine of wild-type mice treated as indicated. Proximal 12 cm of the small intestine used for *N. brasiliensis* and distal 12 cm for succinate. TA, nontuft transit amplifying cell. (**B** to **D**) Flow cytometric analysis of small intestinal enteroids from wild-type mice treated for 7 days as indicated. (B) Representative gating of tuft cells and KIT expression. (C) Frequency of tuft cells (DCLK1^+^ CD24^+^). (D) MFI of KIT on tuft cells. (**E**) Immunofluorescent imaging of KIT in proximal small intestine of mice of indicated genotypes treated as indicated. (**F**) MFI of KIT on tuft cells (DCLK1^+^) from images in (E). D, days; DPI = days postinfection. *N.b.*, *N. brasiliensis*. In graphs, each data point represents a biological replicate. Data are representative of at least three [(A) and (B)] or at least two (E) experiments or pooled from at least two [(C) and (D) and (F)] experiments. Statistics: ordinary one-way ANOVA [(C) and (D)] or two-way ANOVA (F).

Next, we used immunofluorescence to label KIT protein in the small intestine of mice given succinate in their drinking water, infected with *N. brasiliensis* or *H. polygyrus bakeri*, or injected with recombinant IL-25 (rIL-25), all of which are known to induce IL-4/13. Consistent with our *Il4ra*-deficient tuft cell sequencing data, tuft cell KIT was up-regulated when compared to naïve littermates (Fig. 3A and fig. S3D). During type 2 inflammation, KIT is present on tuft cells, Paneth cells, and some DCLK1^−^ cells just above the crypt base, which could be immature tuft cells that do not express detectable DCLK1 or LGR5^−^ stem cells that reside in this region (*43*). In the villi, KIT was detectable only on tuft cells, and all tuft cells were marked by KIT. Using enteroids, we found that IL-13 (10 ng/ml) was sufficient to induce KIT on >95% of tuft cells (Fig. 3, B to D).

We also tested if IL-4/13 signaling is necessary for KIT up-regulation on tuft cells using *Il4ra*^*fl*/fl^;*Vil1-Cre* mice and littermate controls infected with *N. brasiliensis* or given succinate-treated water for 7 days. As previously reported, tuft cells failed to expand in the absence of *Il4ra* (fig. S3E) (*2*). Compared to controls, a smaller proportion of the remaining tuft cells expressed detectable KIT and the median fluorescence intensity (MFI) of KIT on these cells remained at baseline in both helminth-infected and succinate-treated mice (Fig. 3, E and F, and fig. S3F). Together, IL-4/13 is both necessary and sufficient to induce KIT on tuft cells and perhaps Paneth cells and other crypt epithelial cells.

### KIT promotes tuft cell hyperplasia during helminth infection

We began testing the function of epithelial KIT in vitro by using enteroids. We treated enteroids derived from KIT10;Vil1-*Cre*^+^ mice and littermate controls with rIL-13 and found equivalent tuft cell frequencies (fig. S4, A and B). Although an excellent model, enteroids do not fully recapitulate the complexities of the in vivo environment. Notably, enteroid cultures eliminate coordination between the SIE and nonepithelial cells (*44*), thereby disrupting endogenous growth factor gradients, which are replaced with high concentrations of exogenous growth factors such as EGF (*45*). Moreover, enteroids lack spatial niches such as fully developed villi and basal tensile interactions that influence SIE differentiation (*46*). Therefore, we returned to in vivo models to further test the function of epithelial KIT.

**Fig. 4.**
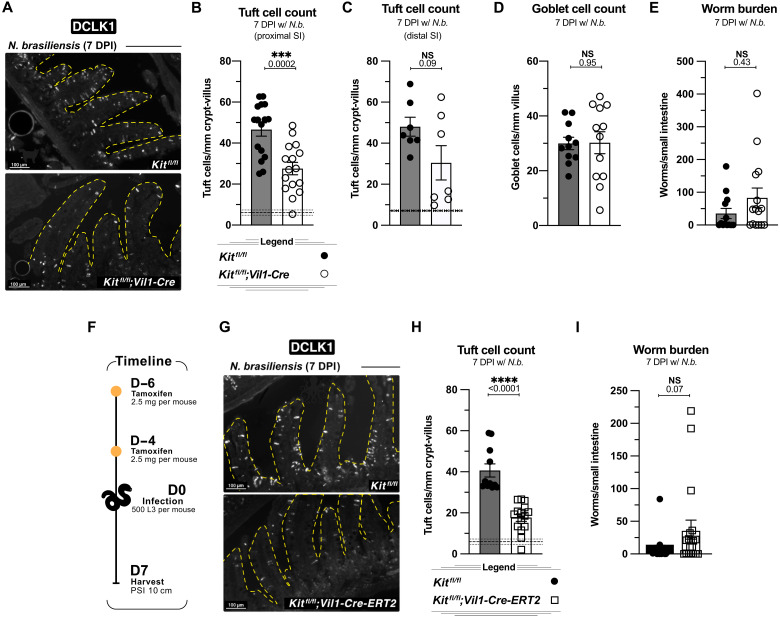
KIT promotes tuft cell hyperplasia during helminth infection. (**A** to **E**) Analysis of *Kit^fl/fl^* and *Kit^fl/fl^;Vil1-Cre* mice 7 days post–*N. brasiliensis* infection. (A) IF staining of tuft cells (DCLK1^+^) in proximal small intestine. (B) Quantification of tuft cells from imaging in (A). (C) Quantification of distal (last 12 cm before the cecum) tuft cells from mice included in (B). (D) Quantification of goblet cells by IF staining (WGA^+^). (E) Total worms counted in the small intestine. (**F** to **I**) Analysis of *Kit^fl/fl^* and *Kit^fl/fl^;Vil1-Cre-ERT2* mice 7 days post–*N. brasiliensis* infection. (F) Experimental schematic. (G) IF staining of tuft cells (DCLK1^+^) in proximal small intestine. (H) Quantification of tuft cells from imaging in (G). (I) Total worms counted in the small intestine. In graphs, each data point represents a biological replicate; thick dashed line represents homeostatic tuft cell baseline calculated from a large cohort of unmanipulated wild-type mice with ±1 SEM (thin dashed line). Data are representative of at least three experiments [(A) and (G)] or pooled from at least three experiments [(B) to (E) and (H) to (I)]. Statistics: unpaired *t* test [(B) to (D) and (H)], Mann-Whitney test [(E) and (I)].

We infected KIT10;*Vil1-Cre* mice and littermate controls with *N. brasiliensis* for 7 days and quantified tuft cells in the proximal small intestine by immunofluorescence microscopy. KIT was absent from all epithelial cells in Cre^+^ mice (fig. S4C), and these mice had a partial (~35%) yet significant defect in tuft cell hyperplasia (Fig. 4, A and B). This defect was more pronounced in the proximal small intestine—where the worms reside—than in the distal small intestine (Fig. 4C). Goblet cell hyperplasia, SI length, and total body weight were unaffected (Fig. 4D and fig. S4, D and E). We also did not find a change in worm burden, indicating that although they were reduced, tuft cell hyperplasia and associated type 2 inflammation remained above the threshold required for worm clearance (Fig. 4E).

To begin to define when KIT is needed and to minimize potential compensatory effects induced by deleting SIE KIT constitutively, we used *Vil1-Cre-ERT2* mice. Cre was activated 6 and 4 days before infection by tamoxifen gavage to acutely delete KIT (Fig. 4F and fig. S4F). Here, tuft cell hyperplasia was reduced ~44% (Fig. 4, G and H). As before, SI length and body weight were unchanged (fig. S4, G and H). There was a trend toward increased worm burden, although not statistically significant (Fig. 4I).

During succinate treatment, type 2 inflammation is predominantly localized to the distal SI, and tuft cell hyperplasia does not reach the magnitude observed in helminth-infected mice (*18*). In mice given succinate, the constitutive deletion of KIT with *Vil1*-Cre caused no significant defects (fig. S4, I to L); however, there was a significant defect in tuft cell hyperplasia following inducible deletion (*Vil1-Cre-ERT2*) (fig. S4, M and N). Thus, KIT contributes to IL-4/13–induced tuft cell hyperplasia, especially in the proximal SIE of a helminth-infected mice, and this dependency is more pronounced following acute KIT deletion.

### KIT is largely dispensable for tuft cell effector function, survival, and migration

KIT signaling has been linked to cell division, survival, migration, and effector function across various cell types. Defects in any of these processes could be nonmutually exclusive explanations for the reduction in hyperplasia when tuft cells lack KIT. For example, decreased survival would directly reduce tuft cell frequency, while less IL-25 and LTC_4_ release would indirectly reduce tuft cell hyperplasia by lowering ILC2 secretion of IL-13.

To test if KIT supports tuft cell production of LTC_4_ in vitro, we cultured epithelial monolayers from KIT10;*Vil1-Cre^+^* mice overnight with or without rIL-13 and then stimulated for 30 min with ionomycin or vehicle to induce LTC_4_ synthesis and release. rIL-13–stimulated monolayers produced more LTC_4_, but there was no significant decrease in LTC_4_ production from KIT-deficient monolayers, suggesting that KIT is dispensable for LTC_4_ production (fig. S5A).

**Fig. 5.**
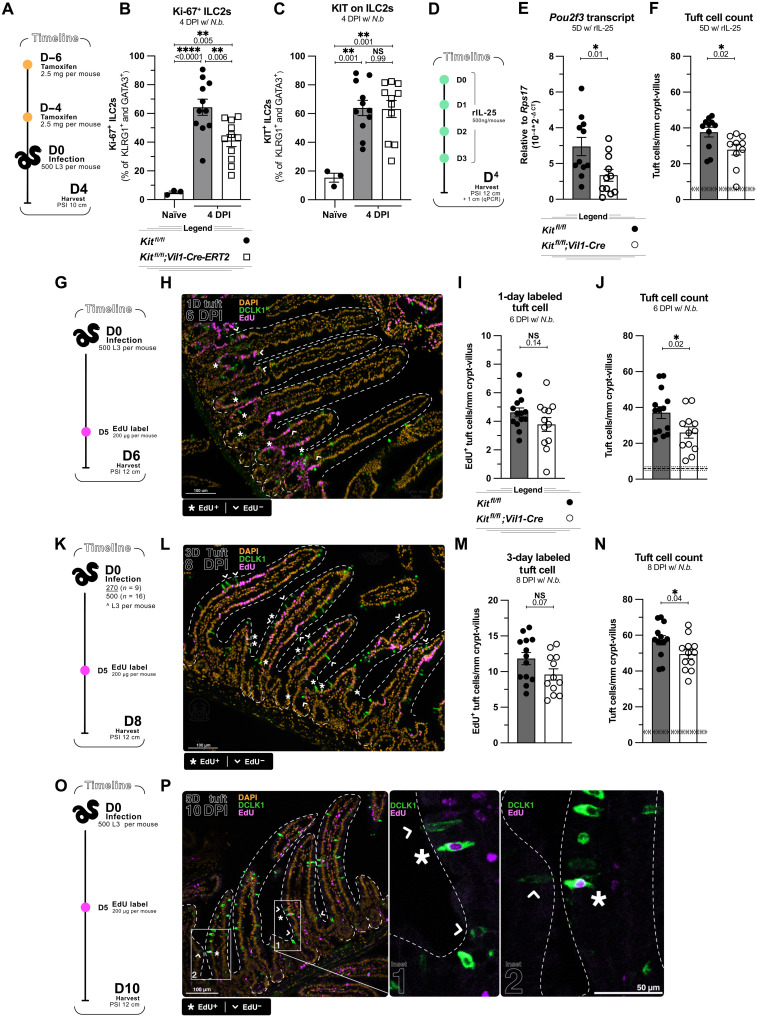
KIT promotes the generation of tuft cells rather than their effector function. (**A** to **C**) Analysis of *Kit^fl/fl^* and *Kit^fl/fl^;Vil1-Cre-ERT2* littermates 4 days after *N. brasiliensis* infection. (A) Experimental schematic. (B) Flow cytometric quantification of Ki-67^+^ ILC2s. (C) KIT quantification. (**D** to **F**) Analysis of *Kit^fl/fl^* and *Kit^fl/fl^;Vil1-Cre* littermates after 4 daily injections of recombinant IL-25. (D) Experimental schematic. (E) Quantitative PCR analysis of *Pou2f3* in 1 cm of proximal small intestinal tissue. (F) Quantification of tuft cells (DCLK1^+^) by IF. (**G** to **J**) Analysis of *Kit^fl/fl^* and *Kit^fl/fl^;Vil1-Cre* littermates 6 days after *N. brasiliensis* infection and 1 day after EdU injection. (G) Experimental schematic. (H) IF imaging of DAPI, DCLK1, and EdU in proximal small intestine. (I) Quantification of EdU^+^ DCLK1^+^ tuft cells from (H). (J) Quantification of total DCLK1^+^ tuft cells from (H). (**K** to **N**) Analysis of *Kit^fl/fl^* and *Kit^fl/fl^;Vil1-Cre* littermates 8 days after infection and 3 days after EdU injection. (K) Experimental schematic. (L) IF imaging of DAPI, DCLK1, and EdU in proximal SI. (M) Quantification of EdU^+^ DCLK1^+^ tuft cells from (L). (N) Quantification of total DCLK1^+^ tuft cells from (L). (**O** to **P**) Analysis of wild-type mice 10 days after infection and 5 days after EdU injection. (O) Experimental schematic. (P) IF imaging of DAPI, DCLK1, and EdU. * = examples of EdU^+^ tuft cells; arrowheads = examples of EdU^−^ tuft cells. Each data point represents a biological replicate; thick dashed line indicate homeostatic tuft cell baseline from unmanipulated wild-type mice with ±1 SEM (thin dashed line). Data are representative of two (P) or three experiments [(H) and (L)] or are pooled from at least two experiments [(B), (C), (E), (F), (I), (J), (M), and (N)]. Statistics: unpaired *t* test [(E), (F), (I), (J), (M), and (N)] ordinary one-way ANOVA [(B) and (C)].

Because we have never been able to measure IL-25 release from tuft cells, and LTC_4_ release is difficult to measure quantitatively in vivo, especially at early time points, we used downstream ILC2 activation as a proxy*. N. brasiliensis* larvae arrive in the small intestine about 2 days postinfection (DPI) and ILC2s up-regulate PD-1, Ki-67 and KIT starting 4 DPI (fig. S5, B to F). Using KIT10;*Vil1-Cre-ERT2* mice, we found that KIT and PD-1 up-regulation were unchanged in KIT-deficient mice, while Ki-67 was partially decreased (Fig. 5, A to C, and fig. S5G). This result suggested that KIT might influence the release of tuft cell effectors that specifically regulate ILC2 replication. At the same time, it is difficult to identify proximal causes of defects in the feed-forward tuft-ILC2 circuit. For example, a defect in tuft cell differentiation from days 2 to 3 of infection could also result in reduced ILC2 activation on day 4 since there would be fewer tuft cells available to produce IL-25 and LTC_4_. Thus, we sought to isolate epithelial effects by normalizing ILC2 activation. After four daily rIL-25 injections to hyperstimulate ILC2s, *Il13* expression was induced at least as well in KIT-deficient mice as in littermate controls, yet *Pou2f3* transcript and tuft cell count were again decreased in the absence of KIT (~25%; Fig. 5, D to F, and fig. S5H). Thus, while our results do not definitively rule out a role for KIT in tuft cell effector functions, KIT can influence tuft cell abundance even when ILC2 activation is normalized.

Within the epithelium, the extent of tuft cell hyperplasia is determined both by the generation of new tuft cells and by their survival within villi. To determine at which stage KIT supports tuft cell hyperplasia, we performed in vivo pulse-chase experiments using EdU, which is stably incorporated into the DNA of dividing cells. Because *N. brasiliensis* reach the intestine around 2 DPI, we injected KIT10;*Vil1-Cre* mice and littermate controls with EdU 5 DPI, a time point when tuft cells are actively expanding and tuft cell hyperplasia is just emerging at the base of villi. One (6 DPI) or three (8 DPI) days later, we quantified total EdU^+^ DCLK1^+^ tuft cells (Fig. 5, G to N). Although not statistically significant (*P* = 0.14 and 0.07, respectively) compared to littermate controls, we found an ~18% decrease in EdU^+^ tuft cells in KIT-deficient mice at both time points. Since the defect was already apparent after the 1-day chase period and did not increase 2 days later, we hypothesized that KIT is more likely to promote the generation of new tuft cells than the survival of mature tuft cells.

Notably, while analyzing these pulse-chase experiments, we found that EdU-labeled tuft cells were always found closer to the crypts than other EdU-labeled SIE cells, suggesting that tuft cells migrate up the villi more slowly and thus have a longer life span than other migrating SIE cells. After a 5-day chase, tuft cells were the only EdU-labeled cells found in lower regions of the villi (Fig. 5, O to P). In sum, while our experiments did not completely rule out a role for KIT in tuft cell survival or effector function, they were consistent with a role for KIT during tuft cell differentiation.

### KIT is required after lineage commitment but before complete maturation of tuft cells

To further assess when KIT is needed during the tuft cell lifecycle, we turned back to genetic approaches. Previous lineage-tracing experiments demonstrated that SI epithelial cells expressing *Dclk1* always become tuft cells, except in very rare exceptions when a “ribbon” of DCLK1^−^ traced cells appears in a single villus (*47*). We found the same is true for *Pou2f3* using *Rosa26::STOP^fl/fl^::zsGreen*;*Pou2f3^Cre-ERT2/+^* mice given tamoxifen; except for very rare exceptions, lineage-traced cells were DCLK1^+^ (fig. S6, A and B). Thus, it appears that both *Dclk1-Cre* and *Pou2f3-Cre-ERT2* overwhelmingly target cells that have exclusively committed to a tuft cell fate.

**Fig. 6.**
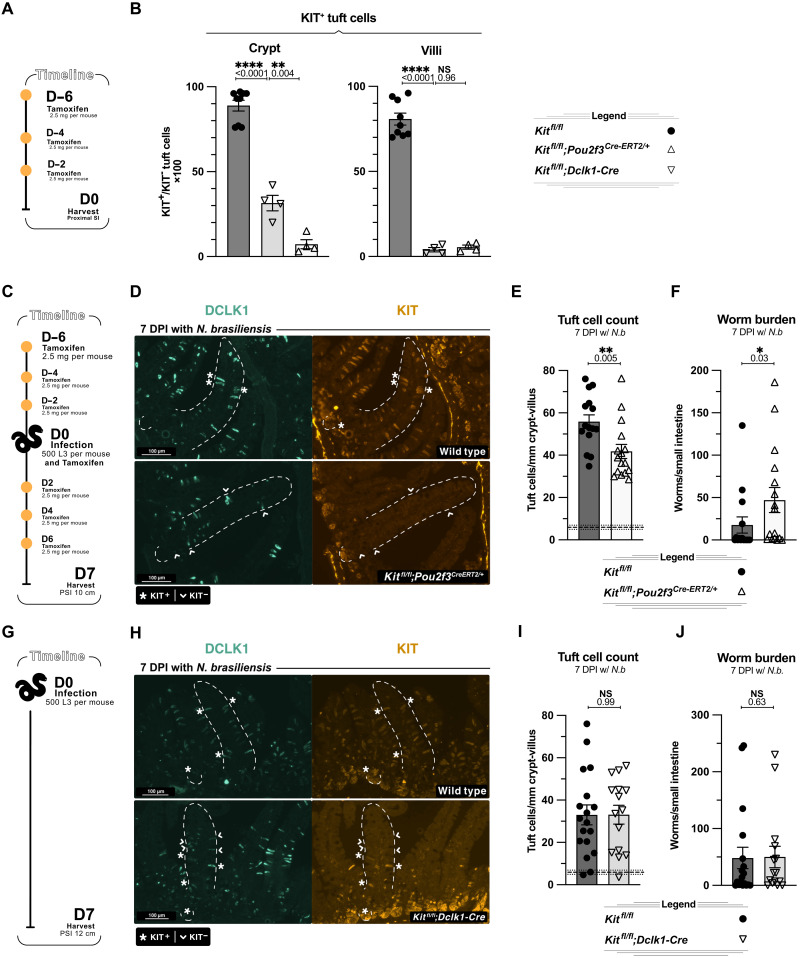
KIT is required early in committed tuft cells to support hyperplasia. (**A** and **B**) IF imaging analysis of naïve *Kit^fl/fl^*, *Kit^fl/fl^;Pou2f3^Cre-ERT2/+^* and *Kit^fl/fl^;Dclk1-Cre* mice. (A) Experimental schematic. (B) Quantification of KIT^+^ tuft cells (DCLK1^+^) at indicated location. (**C** to **F**) Analysis of *Kit^fl/fl^* and *Kit^fl/fl^;Pou2f3^Cre-ERT2/+^* littermates 7 days post–*N. brasiliensis* infection. (C) Experimental schematic. (D) IF staining of DCLK1 and KIT in proximal small intestine. (E) Quantification of tuft cells (DCLK1^+^) from imaging in (D). (F) Worms counted in the small intestine. (**G** to **J**) Analysis of *Kit^fl/fl^* and *Kit^fl/fl^;Dclk1-Cre* littermates 7 days post–*N. brasiliensis* infection. (G) Experimental schematic. (H) IF staining of DCLK1 and KIT in proximal small intestine. (I) Quantification of tuft cells (DCLK1^+^) from imaging in (H). (J) Worms counted in the small intestine. In graphs, each data point represents a biological replicate; thick dashed line represents homeostatic tuft cell baseline calculated from a large cohort of unmanipulated wild-type mice with ±1 SEM (thin dashed line). Data are representative of at least three experiments [(D) and (H)] or pooled from at least two experiments [(B), (E) and (F), and (I) and (J)] Statistics: unpaired *t* test [(E) and (I)] ordinary one-way ANOVA (B) Mann-Whitney test [(F) and (J)].

At the same time, we found a key difference between these two CRE drivers. When we gave KIT10;*Pou2f3^Cre-ERT2/+^* mice tamoxifen every other day for 6 days and then quantified KIT^+^ tuft cells by immunofluorescence and flow cytometry (Fig. 6A), we could not detect KIT on any tuft cells, including those newly generated in the crypts (Fig. 6B and fig. S6, C and D). In contrast, in KIT10;*Dclk1-Cre* mice, we found that many newly generated DCLK1^+^ tuft cells in the crypt and villus base still expressed KIT, while all tuft cells in the upper villi were KIT deficient (Fig. 6B and fig. S6, C and D).

This distinction provided the opportunity to selectively test the function of KIT on committed but newly generated tuft cells. We therefore combined *Pou2f3-* and *Dclk1-*mediated KIT deletion with *N. brasiliensis* infection. Because of the continuous generation of tuft cells, we dosed *Pou2f3-Cre-ERT2* mice with tamoxifen every other day 6 days before infection and during the infection (Fig. 6C). In KIT10;*Pou2f3^Cre-ERT2/+^* mice, KIT was again deleted from all tuft cells, and as in complete epithelial KIT deletion by *Vil1-Cre*, there was a defect in tuft cell hyperplasia and an increase in worm burden even when only tuft cells lacked KIT (Fig. 6, D to F). By contrast, in KIT10;*Dclk1-Cre* mice, we again found that KIT was retained on many crypt tuft cells during *N. brasiliensis* infection, and there was no change in tuft cell hyperplasia or worm clearance (Fig. 6, G to J). As before, neither *Pou2f3-* nor *Dclk1*-mediated KIT deletion altered body weight or SI length (fig. S6, E to I). Collectively, our data support a model in which KIT predominantly supports tuft cell hyperplasia by acting on early tuft cells that reside in the crypts and villus base. Furthermore, the data suggest that KIT is used for tuft cell hyperplasia after *Pou2f3* commitment but before migration to the tops of villi. In other words, KIT supports the differentiation and/or division of an early IL-4/13–sensitive and *Pou2f3*-commited progenitor.

### KIT ligand is constitutively available in the small intestine

To better understand regulation of KIT signaling in tuft cells, we examined expression of *Kitl*, which encodes SCF, the primary ligand for KIT (*48*). By alternative splicing, SCF can be both membrane-bound and secreted and is produced by stromal, endothelial, and some KIT^+^ cells, including POU2F3^+^ taste cells (*26*, *49*). Using our tuft cell mRNA transcript dataset, we observed broad *Kitl* expression across the tissues surveyed (fig. S7A).

**Fig. 7.**
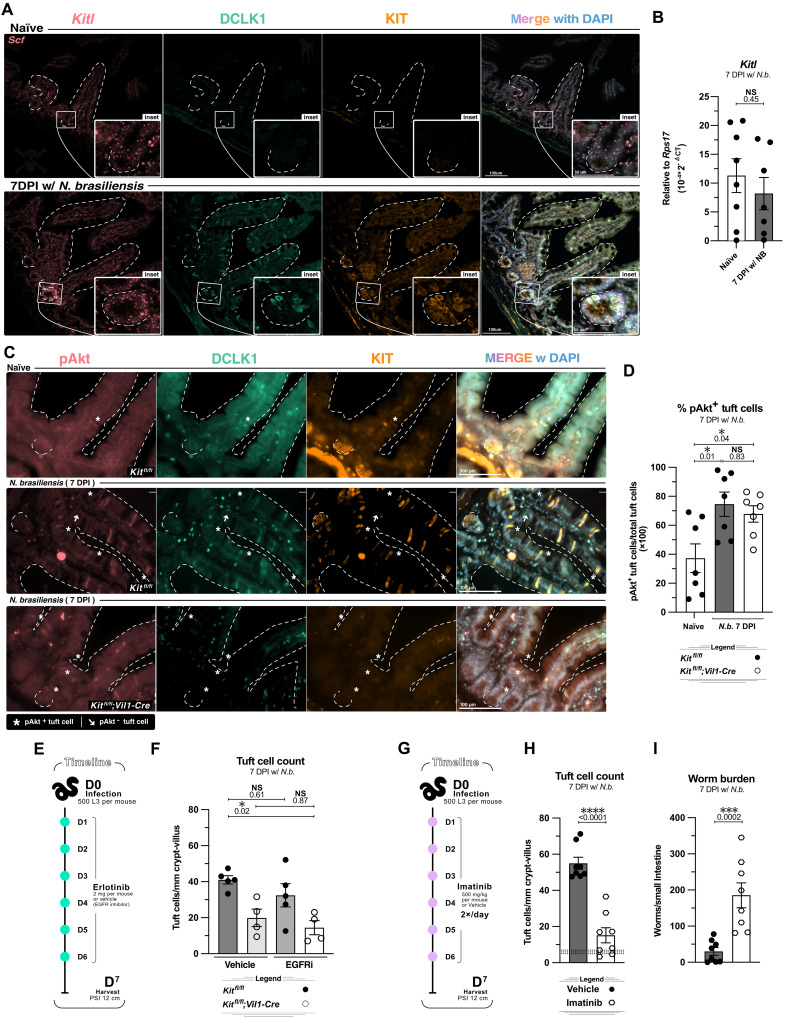
KIT is dispensable for tuft cell Akt phosphorylation, yet KIT inhibition impairs the tuft-ILC2 circuit. (**A** and **B**) Analysis of proximal small intestinal tissue from wildtype naïve mice or mice infected for 7 days with *N. brasiliensis*. (A) RNAscope (in situ hybridization) of *Kitl* and IF staining of indicated proteins*.* (B) qPCR analysis of *Kitl* in 1 cm of small intestine. (**C** and **D**) Analysis of *Kit^fl/fl^* and *Kit^fl/fl^;Vil1-Cre* littermates left untreated or infected for 7 days with *N. brasiliensis*. (C) Representative IF staining of DAPI, KIT, DCLK1, and pAkt in proximal small intestine. (D) Frequency of pAkt^+^ DCLK1^+^ tuft cells from imaging in (C). (**E** and **F**) Analysis of *Kit^fl/fl^* and *Kit^fl/fl^;Vil1-Cre* littermates infected for 7 days with *N. brasiliensis* and treated with EGFR inhibitor (EGFRi) erlotinib or vehicle control. (E) Experimental schematic. (F) Quantification of tuft cells by IF staining (DCLK1^+^). (**G** to **I**) Analysis of wild-type mice infected for 7 days with *N. brasiliensis* and treated with KIT inhibitor imatinib or vehicle control. (G) Experimental schematic. (H) Quantification of tuft cells by IF staining (DCLK1^+^). (I) Worms counted in the small intestine. In graphs, each data point represents a biological replicate. Data are representative of at least two experiments [(A) and (C)] or pooled from at least two experiments [(B), (D), (F), (H), and (I)]. Statistics: unpaired *t* test [(B) and (H)], ordinary two-way ANOVA [(D) and (F)], and Mann-Whitney test (I).

Our experiments using *Pou2f3-Cre-ERT2* and *Dclk1-Cre* indicated a role for KIT signaling while tuft cells are still in the crypt. To assess the spatial distribution of SCF, we performed in situ hybridization for *Kitl* and found that it was expressed throughout the SI epithelium and in many lamina propria cells of both naïve and *N. brasiliensis*–infected mice (Fig. 7A). We next asked whether *Kitl* is up-regulated during helminth-induced inflammation. Whole SI tissue transcript analysis targeting both membrane-bound and secreted forms again revealed high *Kitl* expression at baseline, and there was no further increase with *N. brasiliensis* infection (Fig. 7B). Given the abundance of *Kitl*, it appears that KIT signaling is regulated primarily by variable expression of the receptor rather than the ligand.

### Akt phosphorylation in tuft cells does not require KIT

We also sought to identify downstream targets of KIT signaling in tuft cells. Because KIT is known to induce the PI3K signaling pathway that activates Akt by phosphorylation, and mice lacking raptor (*Rptor*), a downstream target of Akt, have impaired tuft cell hyperplasia (*50*), we used immunofluorescence to detect phosphorylated Akt (pAkt) in the small intestine. We found that tuft cells were the only pAkt-positive cell in the SIE, with ~50% of tuft cells positive for pAkt at baseline (Fig. 7, C and D). The MFI of pAkt in tuft cells did not significantly change in the presence of *N. brasiliensis* or in the absence of KIT. However, the frequency of pAkt^+^ tuft cells increased with infection in a KIT*-*independent manner (Fig. 7, C and D, and fig. S7B).

Many RTKs can induce the phosphorylation of Akt and could therefore act redundantly with KIT. For example, EGF receptor (EGFR) is known to be constitutively phosphorylated in SI tuft cells; thus, we examined the effect of EGFR inhibition with or without KIT deletion (*51*, *14*). KIT10;*Vil1-Cre* mice and littermate controls received the EGFR inhibitor erlotinib or vehicle control daily during *N. brasiliensis* infection (Fig. 7E). While KIT deletion reduced tuft cell hyperplasia as expected, we did not find an effect of erlotinib alone or a further reduction of tuft cells or pAkt in tuft cells when erlotinib was combined with KIT deletion, although we could not quantify the efficiency of EGFR inhibition (Fig. 7F and fig. S7, C to F).

### KIT inhibition blocks tuft cell hyperplasia and helminth expulsion

Previous studies of KIT deficiency or inhibition in helminth infection did not assess effects on tuft cell hyperplasia, nor did they use clinical KIT inhibitors. Imatinib, also known as Gleevec, is another RTK inhibitor that includes KIT among its targets and is used to treat leukemias, systemic mastocytosis, and gastrointestinal stromal tumors. To test how this chemotherapeutic agent might affect intestinal type 2 immunity, we gave imatinib (*52*, *53*) during *N. brasiliensis* infection and harvested at 7 DPI (Fig. 7G). Imatinib induced weight loss, and SI lengthening normally associated with type 2 inflammation was impaired. We also found impaired tuft cell hyperplasia and increased worm burden (Fig. 7, H and I, and fig. S7, G and H). Since *N. brasiliensis* clearance does not require mast cells (*54*), this result further underscored the critical importance of KIT and perhaps other RTKs in the tuft-ILC2 circuit.

## DISCUSSION

Tuft cell hyperplasia—a notable feature of small intestinal helminth infection—promotes worm clearance (*12*, *13*). IL-4/13 signaling in epithelial cells is required for this hyperplasia and, when provided exogenously, also sufficient (*2*). How IL-4/13 signals are translated into tuft cell expansion and whether other signals support this process is largely unknown. Here, we have shown that epithelial KIT is dispensable at homeostasis but during type 2 inflammation cooperates with IL-4/13 signaling to promote tuft cell hyperplasia. The transcript expression of the KIT ligand SCF (*Kitl*) is widespread at baseline and does not change with helminth infection. Instead, IL-4/13 induces expression of KIT itself. Without KIT in tuft cells, tuft cell hyperplasia is reduced, and worm burden is increased. While we were preparing this manuscript, another study reported that the deletion of KIT from the SIE reversed the spontaneous type 2 inflammation and tuft cell hyperplasia induced by the deletion of epithelial *SpiB*, further demonstrating the importance of KIT in tuft cells (*55*).

Our findings provide specific insight into the timing of KIT signaling events that lead to tuft cell hyperplasia. First, we found the same decrease in tuft cell hyperplasia and modest increase in helminth burden, when KIT was deleted from all epithelial cells (*Vil1-Cre* and *Vil1-Cre-Ert2*) or only from tuft cells (*Pou2f3-Cre-Ert2*), indicating that KIT is required after tuft cell lineage commitment and that KIT expression in nontuft epithelial cells (e.g., Paneth) is dispensable in this context. At the same time, there was no change in tuft cell hyperplasia when KIT was deleted with *Dclk1-Cre*. Unlike *Pou2f3-Cre-Ert2*, which deletes KIT from all DCLK1^+^ tuft cells, in KIT10;*Dclk1-Cre* mice, KIT is deleted in the upper villi but still expressed on most tuft cells in the crypts and the lower villi. Also, increasing the “chase” period from 1 to 3 days does not reduce the frequency of EdU-labeled tuft cells. Together, these data demonstrate that KIT predominantly supports the generation of tuft cells in crypts, rather than their survival in the villi.

Although likely KIT independent, we were surprised to observe that the migration of EdU^+^ tuft cells up the villi is slower than other epithelial lineages and that some EdU^+^ tuft cells remain 5 days after EdU injection. Thus, it appears that the turnover of tuft cells is slower than the 2.3 to 4.0 days reported for enterocytes, goblet cells, and enteroendocrine cells (*5*). This slower migration may preferentially support tuft cell accumulation in longer villi of the proximal SI, an effect less apparent in the shorter distal SI villi or in enteroids lacking defined villi.

Whether tuft cells arise exclusively from multipotent progenitors or if committed tuft cells can further divide in crypts, as has been observed in human enteroids (*38*), is not fully resolved. Tuft cell division has, however, never been observed in the villi (*2*). In our experiments, we found a 2.5-fold increase of total EdU-labeled tuft cells from 1 to 3 days post-EdU injection, supporting the model that tuft cells divide after picking up EdU and before exiting crypts. Further studies will be needed to test this model, to delineate the molecular events that lead to tuft cell hyperplasia, and to determine how IL-4/13 promotes goblet cell hyperplasia, as we did not find a role for KIT in the latter process. Because the percent increase in goblet cells is much smaller than tuft cells, perhaps IL-4/13 signals in different progenitors (e.g., DLL1^+^ secretory progenitor) to promote goblet cell differentiation.

We did not study the molecular mechanisms regulating *Kit* expression, but a recent chromatin immunoprecipitation sequencing analysis of POU2F3 found binding near Il4ra, *Il13ra1*, and *Kit*, suggesting direct regulation (*56*). Moreover, we note that there is a canonical signal transducer and activator of transcription 6 (STAT6) binding (TTCTCCAGAA) site just 13 bases from a POU2F3 binding site (ATGCAAAT) in a 3′ enhancer region located between exons 1 and 2 of the *Kit* gene, suggesting the possibility of cooperative binding (*57*, *58*). KIT itself has been suggested to directly dimerize with cytokine receptors such as IL-4RA and to induce downstream signals that synergize with cytokines such as IL-6 (*59*, *60*). IL-4/13 is a necessary upstream signal for KIT induction; however, we have not ruled out potential synergy that could also occur between KIT and the IL-4/13 receptor.

We propose the following model (fig. S7I): First, yet unknown mechanisms lead to the induction of *Pou2f3* in epithelial progenitors, committing them to a tuft cell fate. POU2F3 then induces the expression of *Il4ra* and *Il13ra1*, rendering early tuft cells responsive to IL-4/13; it also induces *Kit* expression at a low level. In the absence of type 2 inflammation, these cells do not receive an IL-4/13 signal; they complete their differentiation into tuft cells and exit the crypts at low frequency. When IL-4/13 is present, however, such as during helminth infection, signaling through the IL-4RA/IL-13RA1 heterodimer leads to STAT6 activation and STAT6 and POU2F3 cooperatively induce *Kit* expression. SCF, which is constitutively available, then signals through KIT to support the further differentiation and/or proliferation of early tuft cells in the crypt, giving rise to tuft cell hyperplasia.

Although our data demonstrate a role for KIT during early tuft cell differentiation, we cannot fully exclude an additional role for KIT in tuft cell effector functions. LTC_4_ secretion in vitro and up-regulation of KIT and PD-1 on ILC2s in vivo were KIT independent. On the other hand, ILC2 replication, as measured by Ki-67, was decreased 4 days post–*N. brasiliensis* infection in the absence of KIT, suggesting decreased tuft cell effector production. While quantification of IL-25 release remains impossible in our hands, the combination of cytokine reporter alleles (e.g., Smart13) with *Kit^fl/fl^* and epithelial CRE should eventually allow us to quantify ILC2 activation at earlier time points before the feed-forward nature of the tuft-ILC2 circuit confounds our interpretations.

KIT is largely dispensable during succinate-induced hyperplasia, which predominantly occurs in the distal small intestine. Even during *N. brasiliensis* infection, KIT deletion had less impact on tuft cell hyperplasia in the distal SI, suggesting that KIT is generally more important in the proximal SI. Inherent differences between helminth- and succinate-induced tuft cell hyperplasia may also contribute. For example, we previously found that only helminth infection requires tuft cell–derived leukotrienes (*14*). How this could integrate with KIT signaling is a topic for future study.

Although RTK signaling is important for all cells, within the SIE, it appears to be uniquely central to tuft cell biology. Previous studies demonstrated that tuft cells are the only SIE cells constitutively marked by an antibody for phosphorylated EGFR (*51*, *14*, *37*). We now show that tuft cells are also the only mature villi epithelial cells that express KIT and the only cells marked by pAkt, a downstream target of both KIT and EGFR. About 40% of tuft cells were pAkt^+^ at homeostasis, and this increased to about 80% during helminth infection, but this was not consistently KIT dependent. These observations, combined with the overall modest impact of epithelial KIT deletion, the evidence for compensation when KIT is constitutively deleted, and the lack of requirement for KIT in enteroids, where EGF is provided at high concentrations, led us to hypothesize that EGFR can compensate for loss of KIT. Our efforts to both delete KIT and inhibit EGFR did not, however, reveal a further loss of pAkt or tuft cell hyperplasia, although we could not validate the effectiveness of our EGFR inhibitor. Additional studies will be needed to determine why Akt and EGFR are uniquely phosphorylated in tuft cells and how their functions overlap.

Another interpretation of our EGFR inhibitor study is that additional tyrosine kinases might be involved. When we used imatinib, which has particular specificity for KIT and the ABL kinase (*61*), we found a nearly complete loss of tuft cell hyperplasia and a defect in helminth clearance. As with all inhibitor studies, it is difficult to determine which cells are the key target(s). Notably, we found that ILC2s also up-regulate KIT during helminth infection; thus, the combined inhibition of ILC2s and tuft cells may explain the more profound phenotype compared to tuft- or epithelium-specific KIT deletion. Nonetheless, our results suggest that KIT inhibitors and RTK inhibitors more generally, which are commonly used in the clinic, can substantially alter type 2 immunity. Relatedly, a complementary study found that the ageusia (loss of taste) reported by many patients taking RTK inhibitors is likely caused by KIT inhibition. Using KIT10 mice, the authors demonstrated a role for KIT in sweet-sensing type II TRCs, which are closely related to tuft cells (*1*, *15*, *62*). Unlike intestinal tuft cells, type II TRCs require KIT homeostatically, and both the number of these cells and the ability to taste sweet ligands were reduced when *Kit* was deleted with *Pou2f3*-Cre-ERT2 or mice were treated with RTK inhibitors.

Responses to both infection and stress broadly require the selective expansion of effector cell types and KIT is often involved. For example, ultraviolet exposure induces KIT-dependent expansion of melanocytes to increase melanin production and thereby provide protection against further DNA damage (*63*). In the context of type 2 immunity, a requirement for KIT on mast cells is well-established (*36*). Here, we have defined an additional role for KIT in type 2 immunity by demonstrating its contributions to tuft cell hyperplasia. Cancer is another setting defined by cellular proliferation. Many tumors express KIT, and gain-of-function *Kit* mutations have been implicated in melanoma, thymic carcinoma, and gastrointestinal stromal tumors (GIST) (*64*). Recent studies have revealed a subset of tuft cell–like carcinomas in all tissues that normally contain tuft cells (intestine, thymus, etc.), and most of these cancers express KIT (*42*). Thus, our findings suggest that while KIT inhibitors approved for GIST and systemic mastocytosis (*65*) may have off-target effects on type 2 immunity, they may be of therapeutic benefit in tuft cell–like cancers.

## MATERIALS AND METHODS

### Experimental animals

Mice aged between 6 and 14 weeks were used for all experiments and age-matched within each experiment. Littermates (e.g., CRE-negative mice) were used as controls in all experiments. Unless otherwise noted, all data represent pooled results from at least two experiments and include both male and female mice. All mice were maintained in specific pathogen–free conditions at the University of Washington and were confirmed to be free of Tritrichomonas by quantitative polymerase chain reaction (qPCR) (*39*). All procedures were conducted within University of Washington Institutional Animal Care and Use Committee guidelines under approved protocols (4390-01). See Table S2 for details about the mouse strains used in this study.

### Generation of KIT10 (**Kit*^*fl/fl*^*) mice

Guide RNAs (IDT) targeting either side of exon 10 of the *Kit* gene were micro-injected into the pronucleus of C57BL/6 embryos together with Cas9 protein and a single-stranded DNA template (IDT) containing 5′ and 3′ homology arms as well as exon 10 and flanking *loxP* sites (see table S3 for guide and template sequences). Embryos were transferred to pseudopregnant dams, and resulting offspring were screened for insertion of both *loxP* sites by Sanger sequencing (see table S3 for PCR primers).

Heterozygous founders were bred to generate homozygous offspring, and further genotyping was performed by PCR (see table S3 for genotyping primers). Homozygous KIT10 (*Kit^fl/fl^*) mice were crossed with various Cre drivers. The Cre-mediated disruption of protein expression was confirmed by flow cytometry or immunofluorescence microscopy.

### Genotyping

Offspring of homozygous KIT10 gene-targeted mice were tested for the KIT10 modification with the 5′ *loxP* primers using a touch-down PCR with annealing temperatures ranging from 65° to 55°C in 1C steps. Amplified products were run on 1.5% agarose (Thermo Fisher Scientific) gels. All mice from the Jackson Laboratories were genotyped according to protocols from the Jackson Laboratories.

### In vivo stimulation and treatment

For succinate experiments, mice were given 150 mM sodium succinate hexahydrate (Thermo Fisher Scientific) ad libitum in drinking water for the indicated amount of time (*39*). IL-4 complexes were generated by incubating 2 μg of mouse recombinant carrier-free IL-4 (BioLegend) with 10 μg of low endotoxin anti-mouse IL-4 antibody (BioLegend) per mouse for 30 min at room temperature. rIL-4 complex or 500 ng of rIL-25 (R&D Systems) was given for four consecutive days intraperitoneally in 200 μl of phosphate-buffered saline (PBS) (*39*). Tissue was harvested on the fifth day. For cellular turnover and pulse-chase experiments, mice were injected intraperitoneally with 200 μg of EdU (Invitrogen) reconstituted in dimethyl sulfoxide (DMSO) and diluted in PBS. Imatinib (Cayman) was resuspended in acetate buffer (pH 5.5) and administered intraperitoneally twice daily, morning and evening, at 50 mg/kg based on starting body weight. Erlotinib (Sigma-Aldrich) was resuspended in DMSO and delivered intraperitoneally once daily at a constant 2 mg per mouse.

### Helminth infections and analysis

*H. polygyrus bakeri* (also known *as H. polygyrus*) and *N. brasiliensis* larvae were raised and maintained as previously described (*66*, *67*). Mice were infected by oral gavage with 200 *H*. *polygyrus bakeri* L3 or subcutaneously at the base of the tail with 500 *N. brasiliensis* L3. Mice were weighed shortly after euthanasia, and the small intestines were flushed and laid out without stretching for total length quantification (*39*). For *N. brasiliensis* infection, the entire small intestine was then flushed with PBS, and the effluent was collected in a petri dish to count worms. SI was fileted open, and all worms remaining in the tissue were enumerated using a dissecting microscope before tissue fixation.

### Tissue immunofluorescence preparation and analysis by microscopy

Intestines, trachea, stomach, thymus, and gallbladder were excised, flushed with PBS, and fixed in 4% paraformaldehyde (PFA) (Electron Microscopy Sciences) with gentle rocking for 3 to 4 hours at 4°C, followed by 30% (w/v) sucrose overnight at 4°C. Samples were then embedded in optimal cutting temperature compound (OCT; Tissue-Tek) and sectioned at 8 μm on a CM1950 cryostat (Leica). Small and large intestine samples were coiled into “Swiss rolls” for embedding.

Unless otherwise noted, immunofluorescent staining was performed in PBS with 1% bovine serum albumin (BSA; Fisher Bioreagents) at room temperature as follows: 1 hour 5% donkey serum, 1 hour with primary antibody (table S4), and 40 min to an 1 hour with secondary antibody against host of primary (table S4). In some cases, primary antibody incubation was overnight at 4°C. For wheat germ agglutinin (WGA) staining slides were washed in 0.1% Tween (Sigma-Aldrich) for the last wash. Then, slides were stained 5 min with 4′,6-diamidino-2-phenylindole in PBS at 1:1000 concentration. EdU staining (Life Technologies) followed the manufacturer’s protocol. After tissue rehydration with PBS and before serum blocking, the Click-it reaction (Life Technologies) was followed by the staining detailed above to combine multiple fluorophores. For pAkt staining, sections were washed with PBS and then permeabilized with 0.05% Triton X-100 (VWR) in PBS (v/v) for 20 min at room temperature. Remaining staining steps were as described above with 3% BSA in PBS (w/v) substituted in washes. Tissue was mounted with VECTASHIELD (Vector Laboratories). Images were acquired on a Zeiss Axio Observer A1, Nikon A1R confocal microscope, Nikon inverted wide-field epifluorescence microscope, or Leica Sp8 microscope with 10× or 20× objectives.

All counts and measurements were calculated using Fiji (ImageJ) software. DCLK1^+^ cells were counted manually and normalized to the distance per crypt-villus axis they occupied (millimeter). Goblet cell counts were calculated by counting WGA^+^ cells in the villi and normalizing to villus length. Paneth cell area was quantified by outlining the LYZ1^+^ area per crypt, and crypts were averaged within image and mice. EdU^+ “^height” was measured from the base of all in-plane crypts to the farthest EdU^+^ nucleus in the villus. MFI of KIT and pAkt was captured in Fiji using the ROI tool to store tuft cell outlines by DCLK1. DCLK1 outlines were applied to KIT or pAkt channel where the mean intensity was measured. These means were normalized to the background signal ofsurrounding epithelium. For each replicate, four 10x images of the Swiss roll were analyzed and at least 20 total villi/crypts were counted, except for pAkt quantification that used 20x images and at least a total of 10 tuft cells per replicate.

### RNAscope

Small intestinal tissue was immediately flushed with ice-cold 4% PFA after harvest and fixed in 4% PFA for an additional 4 hours, gently rocking at 4°C. Following fixation, tissue was submerged in 4% PFA with 30% sucrose for 16 to 24 hours, embedded in OCT, and placed in −80°C. Tissue sections were processed for RNAScope and protein-based antibodies according to the manufacturer’s protocol (Biotechne; table S5) using the protease-free sample pretreatment and as described above.

### Single-cell suspension preparation

For single-cell epithelial preparations from small intestine or colon, tissues were flushed with PBS, and Peyer’s patches were removed, opened longitudinally, and rinsed with PBS (*39*). Tissue was cut into small pieces and incubated either rocking at 37°C for 10 min in Ca^+2^/Mg^+2^-free Hanks’ balanced salt solution (Thermo Fisher Scientific) supplemented with 3 mM EDTA (Invitrogen) and 1 mM Hepes (Thermo Fisher Scientific) or shaking at 37°C for 30 min in RPMI 1640 (Thermo Fisher Scientific) supplemented with 30% fetal bovine serum (FBS) (VMR), 1 mM Hepes, deoxyribonuclease I (0.05 mg/ml; Sigma-Aldrich), and collagenase A (1 mg/ml; Sigma-Aldrich). Tissues were vortexed, and released epithelial cells were passed through a filter. Incubations (10 min) were repeated 3× for a total of 30 min. Supernatants were pooled and washed before staining for flow cytometry.

For small intestinal lamina propria preparations, the first 5 cm of the proximal small intestine were isolated, Peyer’s patches removed, and the tissue opened longitudinally and cut into 1- to 2-cm sections. Sections were transferred into 35-ml ice-cold HBSS (no Ca^+2^/Mg^+2^) containing 10 mM Hepes and shaken vigorously for 30 s. Tissue pieces were filtered through a mesh, stored on ice in RPMI 1640 (Thermo Fisher Scientific) + 5% fetal calf serum (FCS) then transferred into 15-ml prewarmed EDTA [HBSS (no Ca^+2^/Mg^+2^) + 10 mM Hepes + 3 mM EDTA] and shaken for 10 min at 200 rpm at 37°C. This wash was repeated twice (three washes total) to remove the epithelium, with tissue rinsed between washes with 10 ml of prewarmed HBSS (no Ca^+2^/Mg^+2^) + 10 mM Hepes. Following EDTA washes, tissues were digested in 10 ml of prewarmed RPMI 1640 supplemented with 20% FCS (Biowest), collagenase A (1 mg/ml; Sigma-Aldrich), 10 mM Hepes, and deoxyribonuclease I (1 mg/ml; Sigma-Aldrich) and shaken at 200 rpm at 37°C for 30 min. Digested tissues were vortexed, filtered sequentially through a 100- and 40-μm mesh, and washed with ice-cold PBS + 3% FCS. Cells were pelleted by centrifugation (5 min, 1500 rpm), the supernatant was discarded, and the cell pellet washed and stained for flow cytometry.

Enteroids were washed in ice-cold PBS twice and pelleted. Pellets were resuspended in 1× TrypLE (Gibco) diluted with PBS, sheared with a 28-gauge insulin syringe, incubated for 10 min at room temperature, washed, and then stained for flow cytometry.

### Flow cytometry

Single-cell suspensions were stained with Zombie Violet (BioLegend) for live/dead exclusion and then labeled with surface antibodies in PBS + 3% FBS. For antibodies used in this study, see table S4. Except for FLARE25 mice (Fig. 1), cells were fixed and permeabilized after staining for surface markers. For DCLK1 staining, cells were first fixed with IC Fixation buffer (Invitrogen) and then permeabilized. For all other intracellular stains, the eBioscience Foxp3/Transcription Factor Staining Buffer Set was used following the manufacturer’s instructions. Samples were acquired on a Canto RUO or Symphony A3 (BD Biosciences) and analyzed with FlowJo 10 (Tree Star). Debris was excluded with forward scatter (FSC)-A/side scatter (SSC)-A gating, and FSC-A/FSC-H was used to select single-cell events.

### RNA sequencing

Single-cell epithelial suspensions were generated and stained as described above and then 500 tuft cells (CD45^low^ EpCAM^+^ CD24^+^ SigF^+^) sorted directly into lysis buffer from the SMART-Seq v4 Ultra Low Input RNA Kit (Takara). cDNA was generated following the manufacturer’s instructions. Four biological replicates were collected for each genotype. Each biological replicate represents one mouse. Next-generation sequencing and analysis was performed by the Benaroya Research Institute Genomics Core. Sequencing libraries were generated using the Nextera XT library preparation kit with multiplexing primers, according to the manufacturer’s protocol (Illumina), and library quality was assessed using the TapeStation (Agilent). High-throughput sequencing was on HiSeq 2500 (Illumina), sequencing dual-indexed and single-end 58–base pair reads. All samples were in the same run with target depth of 5 million reads to reach adequate depth of coverage.

Sequencing was inspected by FASTQC (v0.11.3) and one sample that failed quality control (QC) was discarded. The following analytic pipeline was managed on the Galaxy platform. Reads were trimmed by 1 base at the 3′ end and then trimmed from both ends until base calls had a minimum quality score of at least 30 (Galaxy FASTQ Trimmer tool v1.0.0). The sequence alignment was performed using STAR aligner (v2.4.2a) with the GRCm38 reference genome and gene annotations from Ensembl release 91. Gene counts were generated using HTSeq-count (v0.4.1). Quality metrics were compiled from PICARD (v1.134), FASTQC (v0.11.3), and HTSeq-count. Raw input from HTSeq-count was normalized in DESeq2. Uniquely mapped Ensembl IDs (genes and noncoding RNAs) with a mean normalized read count of <10 were excluded.

### Monolayer and enteroid culture

Small intestinal crypts were collected from the first 10 cm from the stomach and used to establish two-dimensional (2D) monolayers on Matrigel in 48-well plates or 3D enteroids in Matrigel domes in 24-well plates, as previously described (*14*, *45*). The following modifications were applied: Normacin (100 μg/ml; InvivoGen) was included in the media, R-spondin was replaced with supernatants from R-spondin–expressing L cells, and recombinant Noggin was replaced with supernatants from Noggin-expressing cells. Enteroid medium was replaced on days 3 and 5, and enteroids were either treated with rIL-13 (2.5 ng/ml; PeproTech) or as indicated in figures on days 1, 3, and 5. On day 7, organoids were harvested for passage or analysis.

Monolayers were treated with rIL-13 (20 ng/ml) or vehicle control at time of plating, and medium was replaced after overnight culture. One to 3 hours later, monolayers were stimulated with ionomycin (1 μg/ml) in HBSS containing calcium for 30 min. Supernatants were stored at −80°C before analysis by Cysteinyl Leukotriene Express ELISA (Cayman Chemical) according to the manufacturer’s protocol.

### Reverse transcription and qPCR

Small intestinal tissue was flushed with ice-cold PBS, and 1 cm of tissue was placed in a 2-ml microcentrifuge tube. Tubes were then snap-frozen on dry ice and stored at −80°C. RLT buffer (QIAGEN) was added to snap-frozen tissue and dissociated for 2 min at 60 rpm using TissueLyser II (QIAGEN) with a single 5-mm stainless steel bead (QIAGEN). The RNA was isolated using the Mini Plus RNeasy Kit (QIAGEN) according to the manufacturer’s instructions and reverse-transcribed using SuperScript II (Thermo Fisher Scientific) following the manufacturer’s protocol. cDNA was used as a template for qPCR with PowerUP SYBR Green (Thermo Fisher Scientific) on a Viia7 cycler (Applied Biosystems) (*39*). Transcripts were normalized to *Rps17* (40*S* ribosomal protein S17) expression. See table S3 for primer sequences.

### Statistical analysis

Statistical analysis was performed as noted in figure legends using Prism 10 (GraphPad) software. Graphs show mean values (±SEM). Unless otherwise noted, samples were analyzed by a normality test to determine if they fit a normal distribution and then tested by a two-tailed (unpaired) *t* test. The following were normally distributed: Tuft and goblet cell counts, Paneth cell area, cell proliferation by EdU, SI length, mouse weight, KIT MFI, and EdU-labeled tuft cells. For non-normal distributions such as worm burden, we used the Mann-Whitney two-tailed test. For multiple comparisons, we compared means of each column by a one-way analysis of variance (ANOVA) or by two-way ANOVA. *P < 0.05, **P < 0.01, ***P < 0.001, and ****P < 0.0001.
