# Supplementary material for: KIT supports small intestinal tuft cell hyperplasia
Source: Sci Adv. 2026 Feb 25;12(9):eady0883. doi: 10.1126/sciadv.ady0883 (PMC12935044; doi:10.1126/sciadv.ady0883)
Supplement: Supplementary file 1 — Figs. S1 to S7 Legends for tables S1 to S5 [file sciadv.ady0883_sm.pdf]

Science Advances
AAAS

Supplementary Materials for

KIT supports small intestinal tuft cell hyperplasia

Heber I. Lara *et al.*

Corresponding author: Jakob von Moltke, jmoltke@uw.edu

Sci. Adv. **12**, eady0883 (2026)
DOI: 10.1126/sciadv.ady0883

The PDF file includes:

Figs. S1 to S7
Legends for tables S1 to S5

Other Supplementary Material for this manuscript includes the following:

Tables S1 to S5

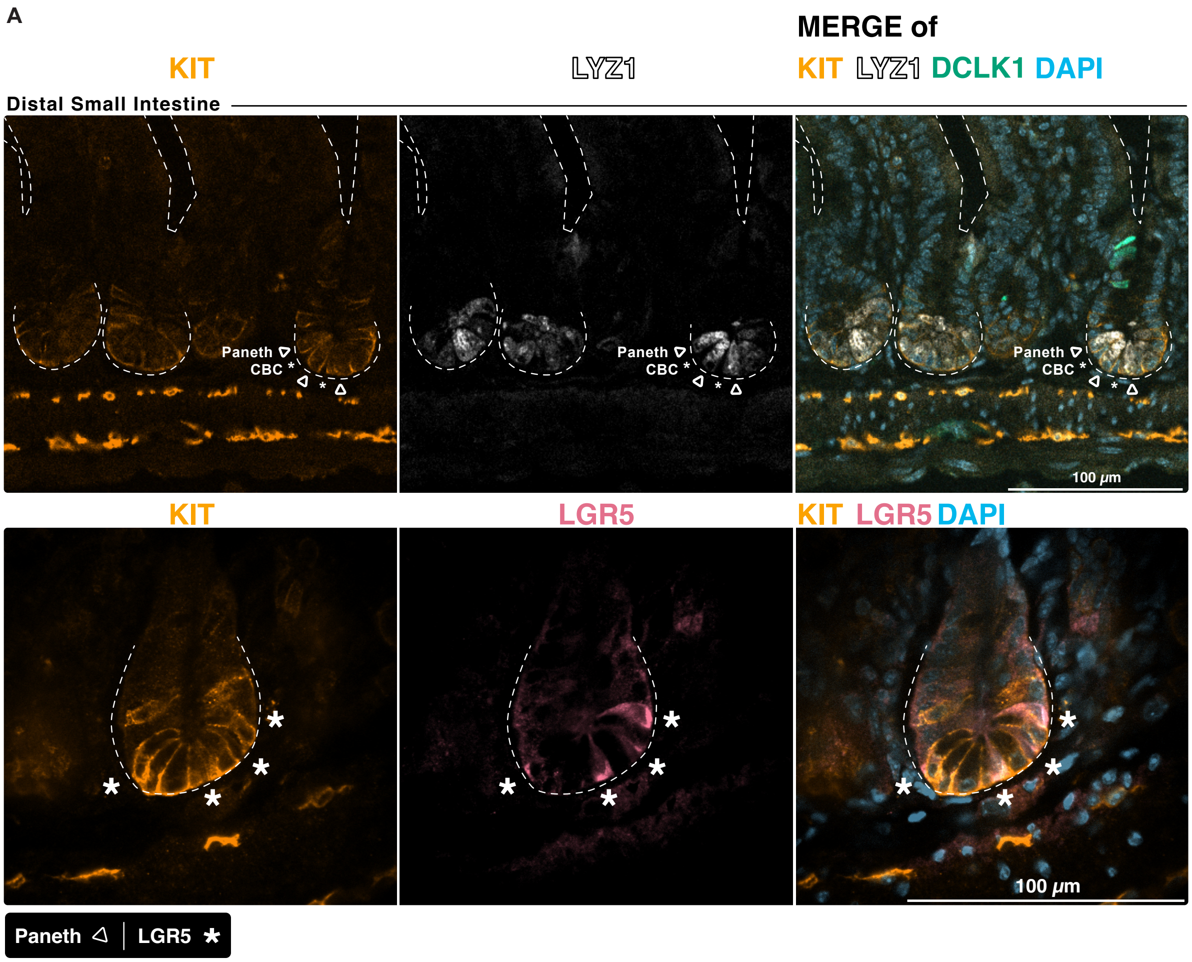

Fig. S1 (Related to Figure 1): (**A**) Immunofluorescent imaging of indicated proteins in distal (last 10 cm) small intestine from unmanipulated wildtype mice. Arrowhead = examples of Lysozyme 1 $(LYZ1)^{+}$ Paneth cells; * = examples of CBC by absence of LYZ1 (upper) or presence of LGR5 (lower). CBC = crypt base columnar cell. Data are representative of 2 experiments.

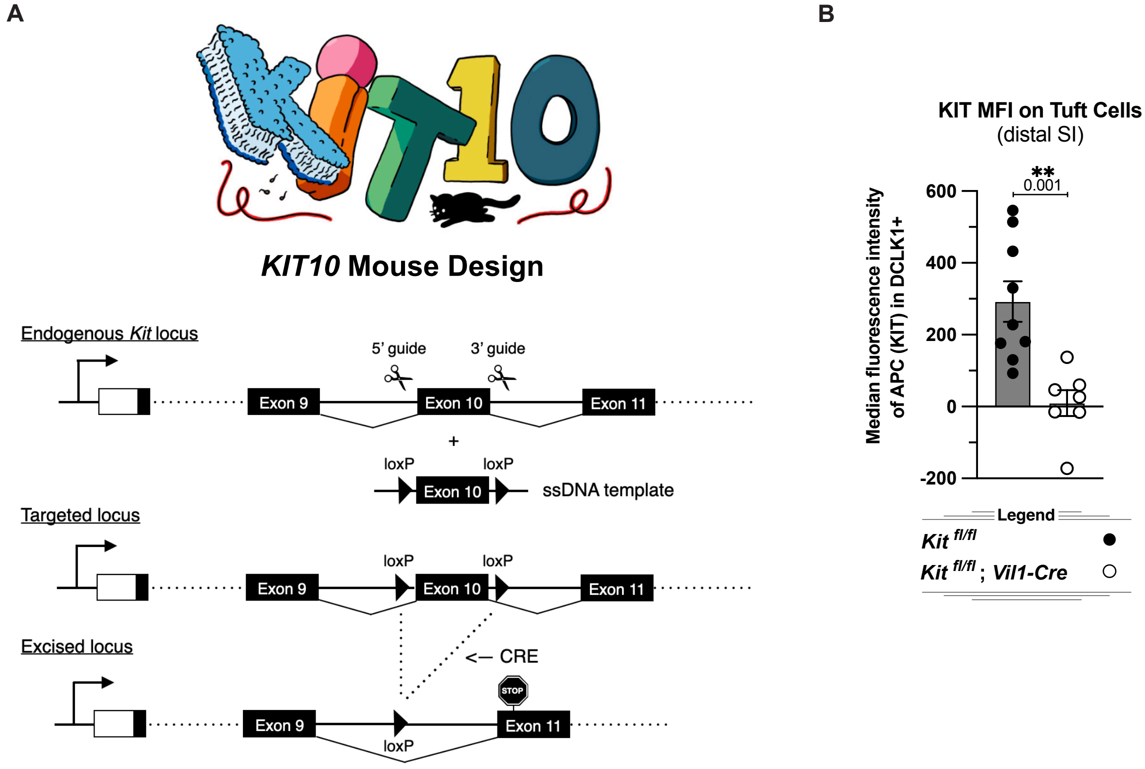

Fig. S2 (Related to Figure 2): (**A**) Diagram of CRISPR/Cas9 targeting strategy to insert *loxP* sites flanking the transmembrane domain-encoding exon 10 of *Kit*. Splicing of exon 9 to exon 11 (if it occurs) gives rise to a stop codon. (**B**) Flow cytometric quantification of KIT MFI on tuft cells (DCLK1^{+} CD24^{+}) from jejunum (10 cm section starting 20 cm before the cecum) of unmanipulated mice of indicated genotypes. Average isotype MFI was subtracted from each sample. Data are pooled from 2 experiments. Statistics: unpaired t-test (B).

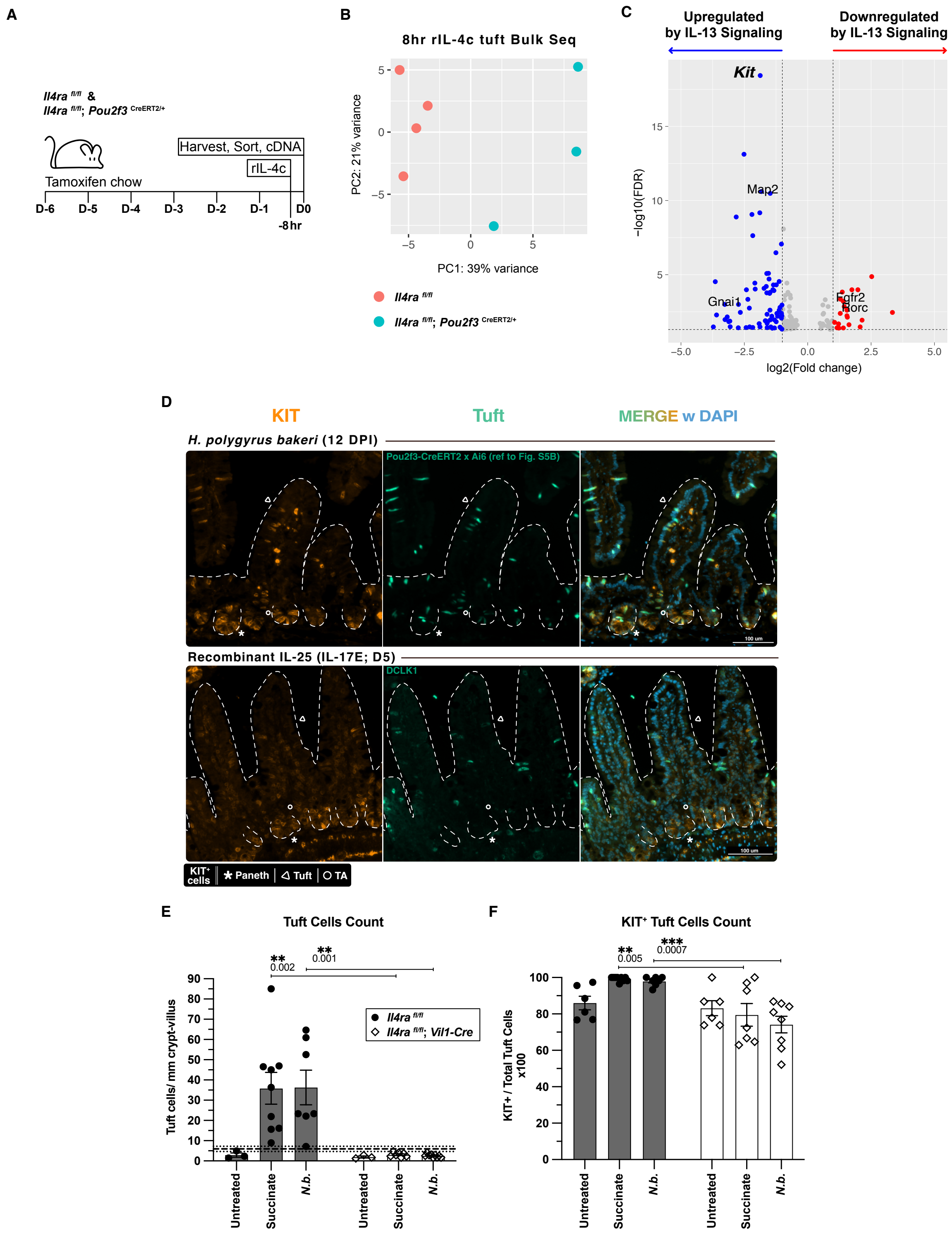

Fig. S3 (Related to Figure 3): (**A**) Experimental schematic for RNA sequencing experiment. (**B**) Principal component analysis of samples generated in (A). (**C**) Volcano plot of differentially

expressed genes from samples generated in (A). (**D**) Immunofluorescent imaging of indicated proteins in small intestine of wildtype mice treated as indicated. TA = non-tuft transit amplifying cell**.** (**E-F**) Quantification of total tuft cells (DCLK1^{+}) (E) and KIT^{+} tuft cells (F) in mice from Figure 3E-F. D = days; DPI = days post infection. *N.b.* = *N. brasiliensis*. In graphs, each datapoint represents a biological replicate; thick dashed line represents homeostatic tuft cell baseline calculated from a large cohort of unmanipulated wild-type mice with ± 1SEM (thin dashed line). Data are from 1 experiment (B-C), representative of 2 experiments (D), or pooled from at least 2 experiments (E, F). Statistics: Ordinary two-way ANOVA (E, F).

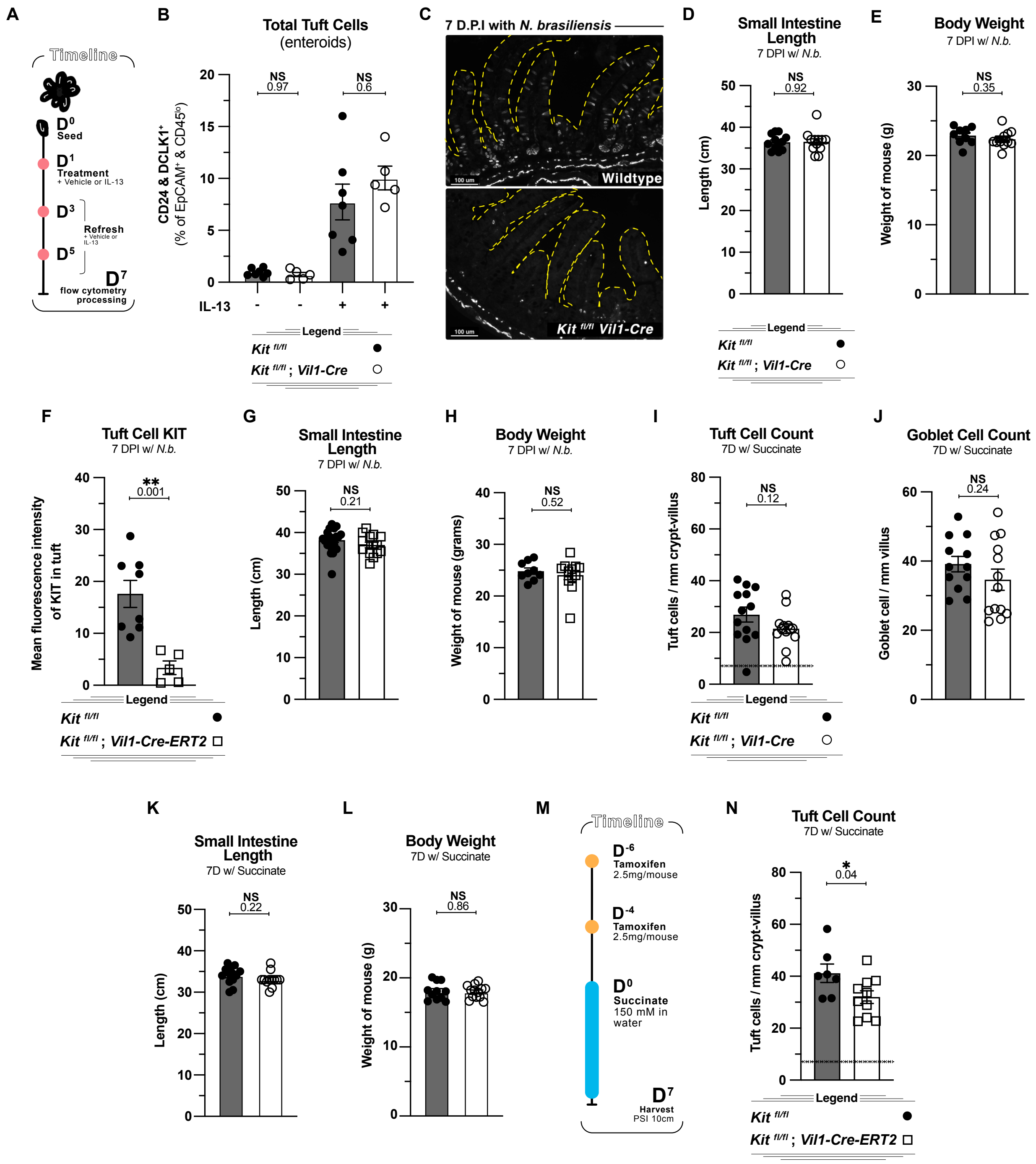

Fig. S4 (Related to Figure 4): (A) Experimental schematic for proximal-derived enteroids in which "refresh" indicates a media change, with DMSO as the vehicle and IL-13 dosed at 10 ng/ml **(B)** Flow cytometric quantification of tuft cells ($DCLK1^+$ $CD24^+$) in enteroids derived from the proximal small intestine of indicated mice and treated as indicated for ~1 week. **(C-E)** Analysis of *Kit$^{fl/fl}$* and *Kit$^{fl/fl}$;Vil1*-Cre littermates 7 days after *N. brasiliensis* infection. (C) Immunofluorescent (I) staining of KIT in proximal small intestine. (D) Small intestinal length. (E) Mouse body weight. **(F-H)** Analysis of *Kit$^{fl/fl}$* and *Kit$^{fl/fl}$;Vil1-Cre-ERT2* littermates 7 days after *N. brasiliensis* infection. (F) Mean fluorescence intensity quantification of KIT on tuft cells

by flow cytometry. **(G)** Small intestinal length. (H) Mouse body weight. (**I-L**) Analysis of *Kit*$^{fl/fl}$ and *Kit*$^{fl/fl}$*;Vil1-Cre* littermates after 7 days of succinate administration. (I) Quantification of tuft cells by IF staining (DCLK1^{+}). (J) Quantification of goblet cells by IF staining (WGA^{+}). (K) Small intestinal length. (L) Mouse body weight. (**M-N**) Analysis of *Kit*$^{fl/fl}$ and *Kit*$^{fl/fl}$*;Vil1-Cre-ERT*2 littermates after 7 days of succinate administration. (M) Experimental schematic. (N) Quantification of tuft cells by IF staining (DCLK1^{+}). D = days. In graphs, each datapoint represents a biological replicate; thick dashed line represents homeostatic tuft cell baseline calculated from a large cohort of unmanipulated wild-type mice with ± 1SEM (thin dashed line). Data are representative of 3 experiments (C) or pooled from at least 2 experiments (B, D-L, N). Statistics: unpaired t-test (D-L, N), ordinary two-way ANOVA (B).

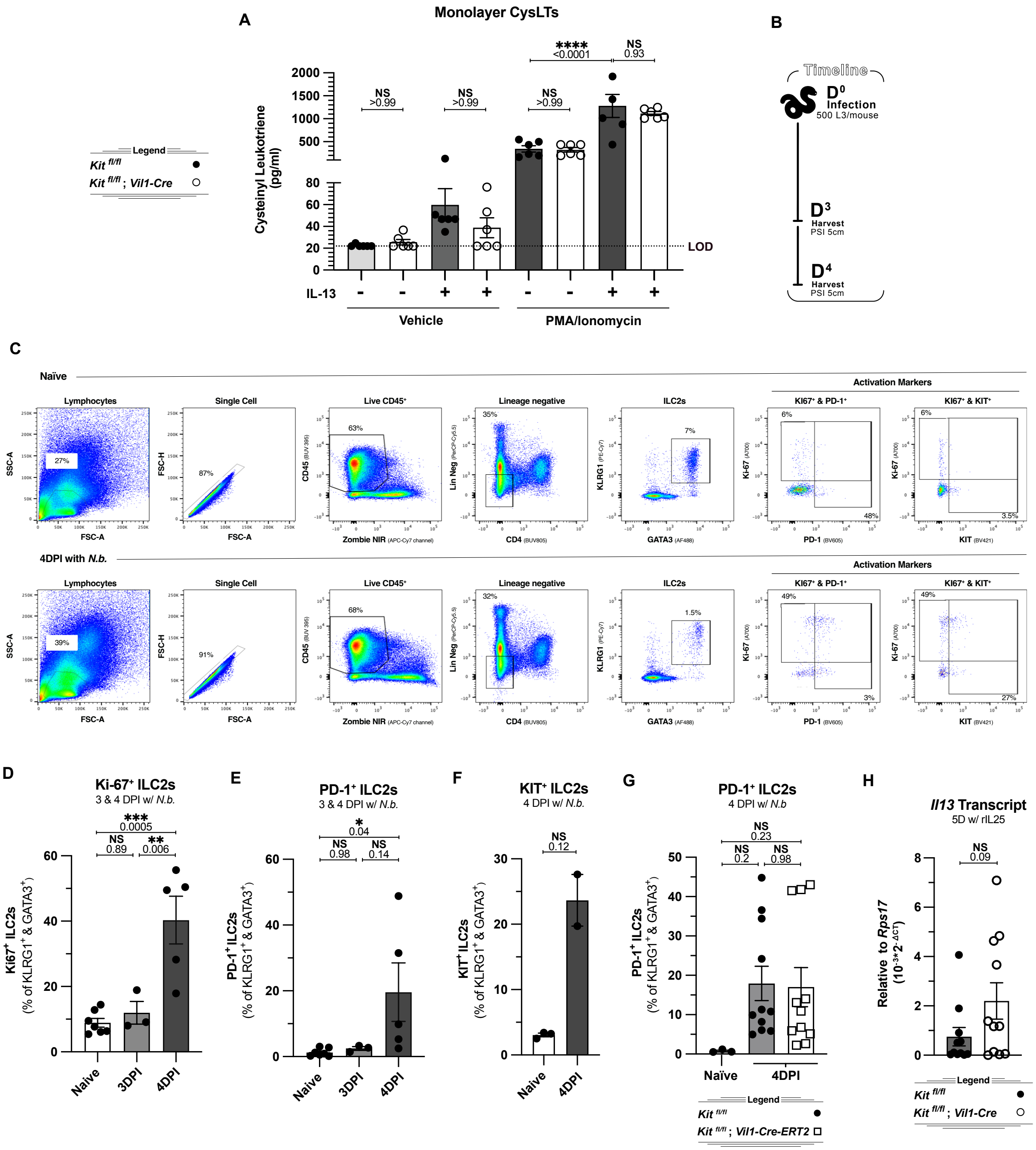

Figure S5 (related to figure 5). (A) Epithelial monolayers derived from proximal intestine of *Kit$^{fl/fl}$* and *Kit$^{fl/fl}$;Vil1-Cre* littermates, cultured in the indicated conditions and analyzed for LTC_4 by ELISA. LOD= limit of detection (22 pg/ml). Readings below 22 were set to 22 pg/ml while those outside the standard curve (>2000 pg/ml) were discarded. **(B-F)** Flow cytometric analysis of lamina propria group 2 innate lymphoid cells (ILC2s) 3 and 4 days after *N. brasiliensis* infection of wild-type mice. (B) Experimental schematic. (C) Representative gating strategy to identify ILC2s in wildtype mice by flow cytometry. Naïve is shown on top and 4 days after N. brasiliensis infection at the bottom. (D) Ki-67 quantification. (E) PD-1 quantification. (F) KIT

quantification. (**G**) PD-1 quantification of lamina propria ILC2s 4 days after *N. brasiliensis* infection of *Kit*$^{fl/fl}$ and *Kit*$^{fl/fl}$*;Vil1-Cre-ERT2* littermates. (**H**) Quantitative PCR analysis of *Il13* in 1cm of proximal small intestinal tissue from rIL-25 stimulated mice. Data are representative of 2 experiments (C) or pooled from at least 2 experiments (A, D, E, F, G, H). Statistics: unpaired t-test (F, H) ordinary one-way ANOVA (D, E, G), ordinary two-way ANOVA (A).

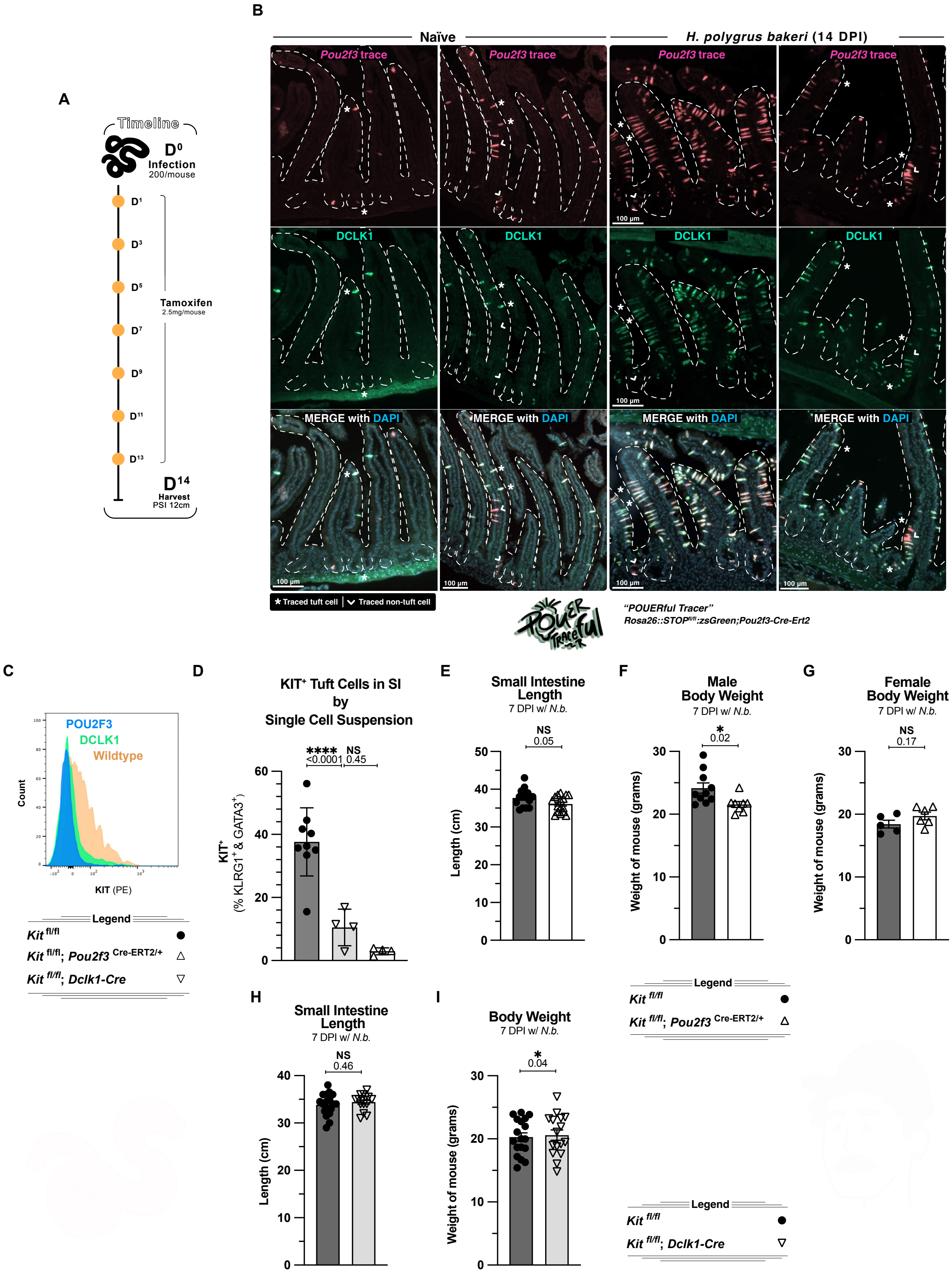

Fig. S6 (related to Figure 6). (A) Experimental schematic for *Pou2f3-Cre-ERT2* lineage tracing

i.e. "POUERful tracer". (**B**) Immunofluorescent (IF) imaging of small intestine from Rosa26:STOP$^{fl/fl}$:zsGreen;*Pou2f3*$^{Cre\text{-}ERT2}$ mice treated as indicated. (**C-D**) Flow cytometric analysis of naïve *Kit*$^{fl/fl}$, *Kit*$^{fl/fl}$*;Pou2f3*$^{Cre\text{-}ERT2/+}$ and *Kit*$^{fl/fl}$*;Dclk1-Cre* mice (C) Representative histogram of KIT expression on tuft cells. (D) Frequency of KIT^{+} tuft cells (DCLK1^{+} CD24^{+}). **(E-G)** Analysis of *Kit*$^{fl/fl}$ and *Kit*$^{fl/fl}$*;Pou2f3*$^{Cre\text{-}ERT2/+}$ littermates 7 days post *N. brasiliensis* infection. Tamoxifen administration as in Figure 6C. (E) Small intestinal length. (F) Male and (G) female mouse body weight. (**H-I**) Analysis of *Kit*$^{fl/fl}$ and *Kit*$^{fl/fl}$*;Dclk1-Cre* littermates 7 days post *N. brasiliensis* infection. (H) Small intestinal length. (I) Mouse body weight. D = days; DPI = days post infection. *N.b.* = *N. brasiliensis*. In graphs, each datapoint represents a biological replicate. Data are representative of 2 experiments (B, C) or pooled from at least 2 experiments (D-I). Statistics: unpaired t-test (E-I), ordinary one-way ANOVA (D).

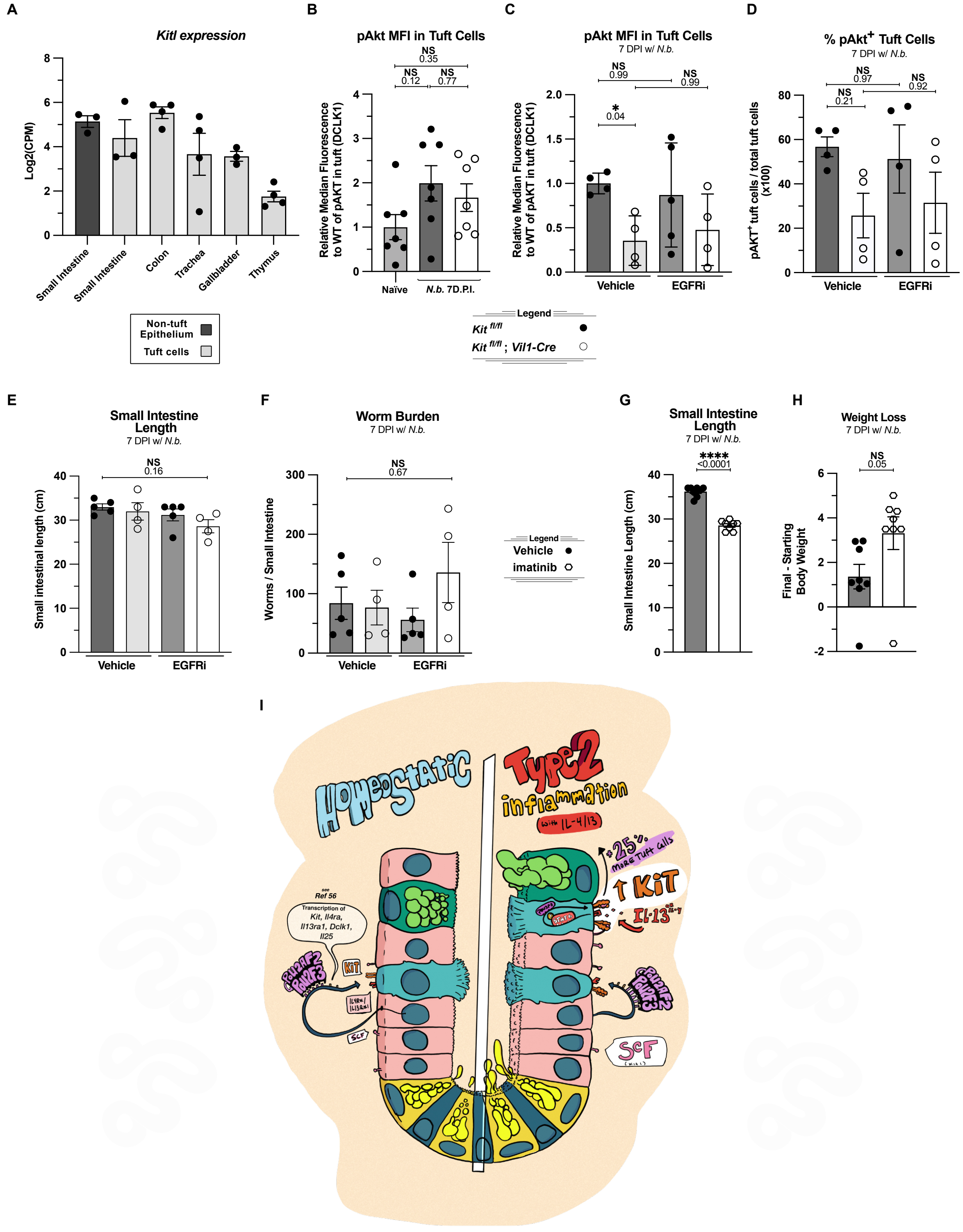

Fig. S7 (Related to Figure 7): (**A**) Normalized RNA transcript reads in tuft cells and non-tuft epithelium sorted from indicated tissue of unmanipulated Flare (*Il25*$^{RFP/RFP}$) mice. Adapted from

Ref #18 (**B**) Mean fluorescence intensity (MFI) by immunofluorescent (IF) imaging of pAKT in tuft cells ($DCLK1^+$) from images in Figure 7C. (**C-F**) Analysis of *$Kit^{fl/fl}$* and *$Kit^{fl/fl}$;Vil1-Cre* littermates infected for 7 days with *N. brasiliensis* and treated with EGFR inhibitor erlotinib or vehicle control. (C) Mean fluorescence intensity (MFI) by IFF imaging of pAKT on tuft cells ($DCLK1^+$). (D) Frequency of $pAKT^+$ $DCLK1^+$ tuft cells. (E) Small intestinal length. (F) Worms counted in the small intestine. (**G-H**) Analysis of wild-type mice infected for 7 days with *N. brasiliensis* and treated with KIT inhibitor imatinib or vehicle control. (G) Small intestinal length. (H) Weight difference from starting to final weight. (**I**) Proposed model where left represents a homeostatic small intestinal epithelial crypt and the right a type 2 inflamed crypt. *N.b. = N. brasiliensis*. DPI = days post infection. In graphs, each datapoint represents a biological replicate. Data are pooled form at least 2 experiments (A-H). Statistics: unpaired t-test (G, H) ordinary two-way ANOVA (B-F).

Table S1. RNA sequencing data of *Il4ra*;*Pou2f3-CreERT2*. List of top differentially expressed genes.

Table S2. Mouselines. A summary of all mouse strains used in this study and their sources.

Table S3. Oligonucleotides. A summary of all DNA oligonucleotides used in this study.

Table S4. Antibodies. A summary of all antibodies used in this study and their sources.

Table S5. Reagents and software. A summary of all reagents and software used in this study and their sources.
